# Regulators of Lysosome Function and Dynamics in *Caenorhabditis elegans*

**DOI:** 10.1534/g3.116.037515

**Published:** 2017-01-24

**Authors:** Kevin Gee, Danniel Zamora, Teresa Horm, Laeth George, Cameron Upchurch, Justin Randall, Colby Weaver, Caitlin Sanford, Austin Miller, Sebastian Hernandez, Hope Dang, Hanna Fares

**Affiliations:** Department of Molecular and Cellular Biology, University of Arizona, Tucson, Arizona 85721

**Keywords:** lysosome, *Caenorhabditis elegans*, coelomocyte

## Abstract

Lysosomes, the major membrane-bound degradative organelles, have a multitude of functions in eukaryotic cells. Lysosomes are the terminal compartments in the endocytic pathway, though they display highly dynamic behaviors, fusing with each other and with late endosomes in the endocytic pathway, and with the plasma membrane during regulated exocytosis and for wound repair. After fusing with late endosomes, lysosomes are reformed from the resulting hybrid organelles through a process that involves budding of a nascent lysosome, extension of the nascent lysosome from the hybrid organelle, while remaining connected by a membrane bridge, and scission of the membrane bridge to release the newly formed lysosome. The newly formed lysosomes undergo cycles of homotypic fusion and fission reactions to form mature lysosomes. In this study, we used a forward genetic screen in *Caenorhabditis elegans* to identify six regulators of lysosome biology. We show that these proteins function in different steps of lysosome biology, regulating lysosome formation, lysosome fusion, and lysosome degradation.

Lysosomes are membrane-bound organelles that serve as the major degradative compartments for endocytic, phagocytic, and autophagic materials targeted for destruction in eukaryotic cells. This degradation is critical to many physiological processes, including processing endocytosed nutrients, downregulating signaling receptors, presenting antigens, killing pathogenic organisms, and degrading normal and abnormal cellular proteins (Dunn 1994; Mullins and Bonifacino 2001; Luzio *et al.* 2005). In addition, lysosomes mediate some cell death pathways and repair damage to the plasma membrane (Rodriguez *et al.* 1997; Reddy *et al.* 2001; Yamashima *et al.* 2003; Fehrenbacher and Jaattela 2005; Gyrd-Hansen *et al.* 2006). Given these diverse activities, it is perhaps not surprising that lysosomal dysfunction has been linked to the progression of many diseases, including a group of ∼50 that are referred to as lysosomal storage disorders (Vellodi 2005; Ballabio and Gieselmann 2009).

Lysosomes are dynamic structures that undergo cycles of fusion and reformation with endocytic, phagocytic, and autophagic organelles. In the endocytic pathway, mature lysosomes fuse with late endosomes to form hybrid organelles using a molecular machinery that includes Rab7, HOPS complex, and SNARES (Luzio *et al.* 2005; Balderhaar and Ungermann 2013; Yasuda *et al.* 2016). Lysosomes are formed, or reformed, from hybrid organelles in complex eukaryotes by the budding of a small nascent lysosome from the hybrid organelle, movement of the nascent lysosome away from the hybrid organelle while maintaining a membrane bridge, and scission of the membrane bridge to release a discrete primary lysosome; molecules destined for lysosomes are concentrated in the nascent lysosome during lysosome formation/reformation (Treusch *et al.* 2004; Bright *et al.* 2005; Thompson *et al.* 2007; Miller *et al.* 2015). Autophagic lysosome reformation following fusion of autophagosomes with lysosomes is similar, perhaps identical, to lysosome formation in the endocytic pathway (Yu *et al.* 2010; Rong *et al.* 2011, 2012). Lysosome formation requires the Mucolipidosis type IV protein TRPML1 (CUP-5 in *Caenorhabditis elegans*), Ca^2+^ efflux from hybrid organelles, and the *C. elegans* small Rab GTPase 2-like UNC-108 protein (Pryor *et al.* 2000; Chun *et al.* 2008; Lu *et al.* 2008; Miller *et al.* 2015); autophagic lysosome reformation is activated by mTOR signaling, and requires clathrin and phosphatidylinositol-4,5-bisphosphate kinases (Yu *et al.* 2010; Rong *et al.* 2012). Primary lysosomes that are formed are thought to undergo cycles of homotypic fusion with each other using the HOPS complex and SNARES, and fission reactions that yield mature lysosomes that can fuse with late endosomes (Ward *et al.* 2000; Wang *et al.* 2003). In addition to all of these membrane dynamics, lysosomal enzymes are targeted from the biosynthetic pathway to lysosomes, a process referred to as lysosome biogenesis (Mullins and Bonifacino 2001; Luzio *et al.* 2003). Much remains to be discovered about molecular regulation of these complex fusion, fission, and reformation reactions in the late endocytic pathway.

We had made P*myo-3*::*ssGFP* transgenic worms in which GFP is secreted into the body cavity when it is attached to a signal sequence and expressed in body wall muscles. The GFP is endocytosed primarily by six scavenger cells called coelomocytes in the body cavities of hermaphrodite worms, and subsequently degraded in lysosomes; P*myo-3*::*ssGFP* worms display diffuse GFP fluorescence in the body cavity and GFP-filled endolysosomal compartments in coelomocytes (Fares and Greenwald 2001a,b; Treusch *et al.* 2004). Mutations in “*cup*” (coelomocyte uptake) genes that reduce endocytic uptake by coelomocytes lead to increased levels of GFP in the body cavity, while mutations that decrease lysosomal function result in increased compartmental GFP levels in coelomocytes (Fares and Greenwald 2001a). A previous pilot mutational screen identified 10 *cup* genes required for uptake of GFP from the body cavity, and a single gene, *cup-5*, which encodes the worm ortholog of mammalian TRPML1, that is required for lysosome function and that we showed is required for lysosome formation/reformation (Fares and Greenwald 2001a,b). In this study, we carried out a forward genetic screen focused on lysosomal phenotypes and identified six additional *cup* genes that function in different aspects of lysosome biology.

## Materials and Methods

### C. elegans strains and methods

Standard methods were used for the growth and manipulation of *C. elegans* (Brenner 1974). Ethyl methane sulfonate mutagenesis was performed on the wild type var. “Bristol” strain GS1912: *dpy-20(e1282)*; *arIs37[*P*myo-3*::*ssGFP*; *dpy-20]* as previously described (Fares and Greenwald 2001a). Briefly, five mutagenized P_0_ parents were placed on a single NGM (nematode growth medium) plate that had been previously seeded with *Escherichia coli* OP50 (Brenner 1974). The F_2_ hermaphrodites from each plate were visually screened using a modified dissection microscope equipped with fluorescence optics (Zeis, Thornwood, NY) for a defect in lysosome function by coelomocytes evidenced by brighter GFP in coelomocytes. Independently isolated mutant F_2_ worms (only one from each plate) were picked onto individual fresh NGM plate (with OP50), and those that yielded viable progeny were kept for further analysis. Strain GS1912 was also used to backcross mutant strains. The wild type var. “Hawaiian” strain was used for single nucleotide polymorphism mapping, as previously described (Davis *et al.* 2005). RNAi was done by the feeding method; control RNAi was done using bacteria carrying the double-stranded RNA generating vector L4440/pPD129.36 (Timmons and Fire 1998). The *C. elegans* strains used in this study are listed in Supplemental Material, Table S1.

### Microscopy

#### Microinjection:

The body cavities of young adult hermaphrodites were microinjected with 1 mg/ml BSA-Alexa Fluor 594 in 1× PBS at room temperature, as previously described (Dang *et al.* 2004; Treusch *et al.* 2004; Schaheen *et al.* 2009). Microinjected worms were allowed to recover on NGM + OP50 plates, and imaging was done after 30 min or 24 hr on worms placed in 6 μl of 9 mM levamisole/1× PBS on a 2.2% agarose pad.

#### Marker analysis:

Young adult wild type and mutant hermaphrodites were placed in 6 μl of 9 mM levamisole/1× PBS on a 2.2% agarose pad for imaging.

#### Microscopy:

Confocal images were taken on a Zeiss LSM510 (Carl Zeiss, Oberkochen, Germany) laser scanning confocal, using LSM imaging software. Image analysis was done using Metamorph software (Molecular Devices, Sunnyvale, CA).

### Molecular methods

Standard methods were used for the manipulation of recombinant DNA (Sambrook *et al.* 1989). Polymerase chain reaction was done using the Expand Long Template PCR System (Roche, Indianapolis, IN) according to the manufacturer’s instructions. All other enzymes were from New England Biolabs (Beverly, MA).

### Whole-genome sequencing

Mutants were first backcrossed four times to the wild-type GS1912 strain. The backcrossed mutants were crossed one more time to GS1912, and 10 independently isolated homozygous mutants were picked from the F_2_ generation. These 10 hermaphrodites were mixed together for genomic DNA isolation for sequencing; this allowed us to identify background mutations that would not be homozygous in all 10 isolates (unless they are closely linked to the *cup* mutation).

*C. elegans* genomic DNA was sequenced by the University of Arizona Genomics Core using shotgun/*de novo* sequencing on an Illumina HiSequation 2000. Bowtie2 was used to map reads from each sample to the *C. elegans* reference WBcel215. samtools mpileup was then used to identify all of the variations from WBcel215. *cup* mutant sequences were compared to the whole genome sequence of GS1912; unique variants each had an average of 147 reads. This identified homozygous mutations in *cup* strains, relative to the parent strain GS1912.

### Protein domain identification

SMART (Simple Modular Architectural Tool) was used for the identification of protein domains (Schultz *et al.* 1998; Letunic *et al.* 2015).

### Statistical methods

The Student’s *t*-test was used to compare average measurements from two samples using a two-tailed distribution (Tails = 2), and a two-sample unequal variance (Type = 2).

### Data availability

All data and reagents are available upon request. The authors state that all data necessary for confirming the conclusions presented in the article are represented fully within the article.

## Results

### Identification of novel cup mutants with lysosomal defects

We mutagenized ∼3000 P_0_ P*myo-3*::*ssGFP* hermaphrodites, and screened ∼70,000 F_2_ progeny for worms that had coelomocytes with brighter GFP, indicative of a delay in the lysosomal degradation of endocytosed GFP in coelomocytes. We identified 20 independent homozygous mutants that gave viable progeny (Table 1). All of the mutations were recessive, since crossing them to the parent wild-type P*myo-3*::*ssGFP* males yielded F_1_ hermaphrodite progeny that did not have a coelomocyte defect.

**Table 1:** List of *cup* genes and homologs

Gene	Worm ORF	Alleles	Mammalian homolog	*S. cerevisiae* homolog
*cup-5*	R13A5.1	*cd9*, *cd10*, *cd12*, *cd18*, *cd19*, *cd20*, *cd21*	TRPML1	None
*cup-12/clh-6*	R07B7.1	*cd7*, *cd14*, *cd15*, *cd34*, *cd39*	CIC-7	GEF1
*cup-13/aexr-2*	C25B8.7	*cd16*, *cd17*	Prokineticin Receptor	None
*cup-14*	Y51H1A.2	*cd31*, *cd32*	PLEKHM1/3	None
*cup-15*	F42A8.3	*cd33*, *cd46*	OSTM1	None
*cup-16*	C34C6.7	*cd50*	None	None
*cup-17/ctns-1*	C41C4.7	*cd49*	Cystinosin	ERS1

**Figure 1:**
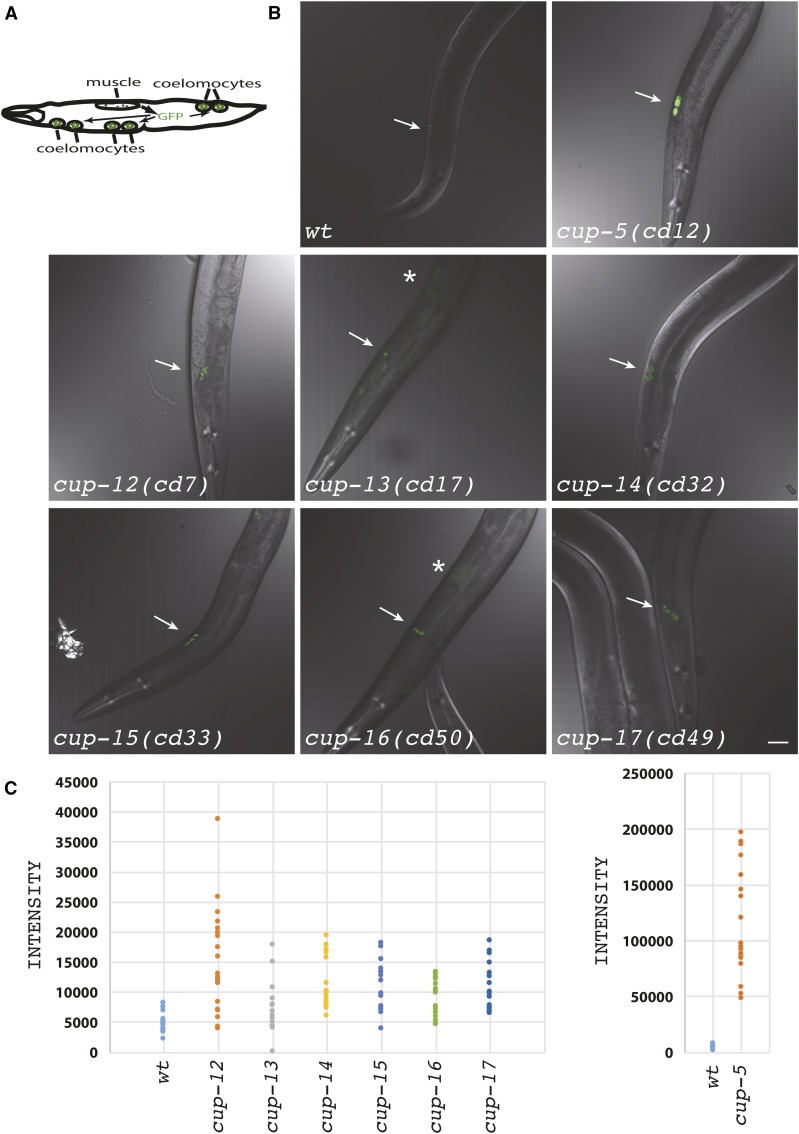
Lysosomal degradation defect of endocytosed GFP. (A) The top left panel is a schematic of an *arIs37[*P*myo-3*::*ssGFP*; *dpy-20]* hermaphrodite showing GFP being secreted from body wall muscle cells into the body cavity, and the endocytosis of this GFP by coelomocytes. Anterior is to the left. (B) Confocal images of the indicated genotypes also carrying the *arIs37[*P*myo-3*::*ssGFP*; *dpy-20]* transgene. All images were taken using the same microscopy settings. Images are shown as an overlay of the GFP fluorescence (green) and DIC (gray) of anterior coelomocytes. Arrows indicate coelomocytes. Asterisks indicate accumulation of GFP in the body cavity. Bar = 20 µm. (C) Quantitation of the GFP intensity in whole coelomocytes of the indicated genotypes also carrying the *arIs37[*P*myo-3*::*ssGFP*; *dpy-20]* transgene. *cup-5* mutant worms are shown separately to adjust for scale.

We used complementation analysis to determine the number of genes that correspond to the 20 mutations. We included the hypomorphic *cup-5(ar465)* allele in this analysis because we had originally identified this mutant on the basis of a similar phenotype in P*myo-3*::*ssGFP* worms (Fares and Greenwald 2001a). Our analysis indicates that we indeed identified several additional viable mutations in *cup-5* (Table 1); these are hypomorphic mutations since null alleles in *cup-5* result in embryonic lethality (Hersh *et al.* 2002; Schaheen *et al.* 2006). In addition to *cup-5*, we identified mutations in six other genes that we referred to provisionally as *cup-12* through *cup-17* (Table 1). *cup-12*, *cup-14*, *cup-15*, and *cup-17* mutants have coelomocytes with brighter GFP than wild-type P*myo-3*::*ssGFP* hermaphrodites; in addition to this brighter GFP phenotype, *cup-13* and *cup-16* hermaphrodites also accumulate GFP in the body cavity, indicating a defect in the uptake of GFP from the body cavity (Figure 1) (Fares and Greenwald 2001a).

We backcrossed alleles of each gene at least four times to the wild-type P*myo-3*::*ssGFP* strain. Imaging of the backcrossed strains confirmed that *cup-12* to *cup-17* mutants had brighter GFP in coelomocytes, indicating a defect in lysosomal transport and/or function in these mutants (Figure 1, A and B). The brighter GFP in coelomocytes of *cup-13* and *cup-16* mutants is an underestimate because of the reduced uptake of GFP into coelomocytes from the body cavity in these mutants (Figure 1, A and B). However, none of the new mutants exhibited as severe a defect as *cup-5* mutants, in terms of the accumulation of GFP in coelomocytes (Figure 1, B and C). Having isolated these new mutants with lysosomal defects, we proceeded to identify the mutated genes.

### Cloning cup-12 to cup-17

We used a similar approach to clone each gene. We first used rapid single-nucleotide mapping to map one mutation in each gene to a chromosome. We then did whole-genome sequencing to identify homozygous mutations that affected predicted protein sequences that mapped to the relevant chromosome for each gene; mutations identified by whole genome sequencing were reconfirmed by regular sequencing, which resulted in a list of candidate genes for each *cup* mutant. To identify the relevant gene/mutation, we used complementation analysis against previously identified alleles of the genes, and/or RNAi of the candidate genes in P*myo-3*::*ssGFP* hermaphrodites, to test for the relevant phenotypes.

#### cup-12:

We isolated five independent alleles of *cup-12* (Table 1). We mapped *cup-12(cd7)* to chromosome V. Whole-genome sequence analysis of *cup-12(cd7)* and of *cup-12(cd15)* worms identified protein-altering mutations that were common in both alleles in three genes on chromosome V, *Y108G3AL.3*, *ZC412.2/gcy-14*, and *R07B7.1*/*clh-6*. We did complementation analysis by crossing *cup-12(cd7)*; P*myo-3*::*ssGFP* males to existing null alleles of *Y108G3AL.3* and *gcy-14*, and to an existing missense allele of *clh-6* (Table S1): only the *clh-6* cross gave F_1_ worms with the *cup-12* mutant bright coelomocyte GFP defect. Indeed, a *clh-6(gk248753)*; P*myo-3*::*ssGFP* strain showed the same Cup phenotype as *cup-12*; P*myo-3*::*ssGFP*. Furthermore, RNAi of *clh-6*, but not of *Y108G3AL.3* or *gcy-14*, yielded the *cup-12* mutant bright-coelomocyte defect in P*myo-3*::*ssGFP* worms. We identified homozygous mutations in *clh-6* in all five *cup-12* alleles; *cd15* introduces a stop codon, while the rest of the alleles have amino acid substitutions in the predicted wild-type protein (Figure S1). Thus, CUP-12 is CLH-6, which is homologous to the lysosomal chloride channel CIC-7 in mammals and to *Saccharomyces cerevisiae* GEF1 (Borsani *et al.* 1995; Bianchi *et al.* 2001; Braun *et al.* 2010).

#### cup-13:

We isolated two independent alleles of *cup-13* (Table 1). We mapped both *cup-13* alleles to the X-chromosome. We had previously identified mutations in a gene we called *cup-11* that mapped to the X-chromosome, and that showed similar defects as *cup-13* (Fares and Greenwald 2001a). Complementation analysis indicated that *cup-11* and *cup-13* were identical. Whole-genome sequence analysis of *cup-13(cd17)* worms identified homozygous, protein-altering mutations in three genes on chromosome X: *col-168*, *aexr-2*, and *F14B8.5*. We did complementation analysis by crossing *cup-13(cd16)*; P*myo-3*::*ssGFP* and *cup-13(cd17)*; P*myo-3*::*ssGFP* males to existing null alleles of *col-168* and *aexr-2*, and to existing missense alleles of *F14B8.5* (Table S1): only the *aexr-2* crosses gave F_1_ worms with the *cup-13* mutant bright-coelomocyte GFP and body cavity GFP defects. Indeed, an *aexr-2(gk533021)*; P*myo-3*::*ssGFP* strain showed the same Cup phenotypes as *cup-13*; P*myo-3*::*ssGFP*. In addition, we identified protein-altering changes in the two independently isolated *cup-13* alleles, and in eight of the nine independently isolated *cup-11* alleles (Figure S2). Thus, CUP-13 is CUP-11, which is AEXR-2, a predicted seven-transmembrane domain, G protein-coupled receptor with homology to Prokineticin and Galanin Receptors in mammals (Floren *et al.* 2000; Ngan and Tam 2008; Lau *et al.* 2015).

#### cup-14:

We isolated two independent alleles of *cup-14* (Table 1). We mapped *cup-14(cd32)* to chromosome II. Whole-genome sequence analysis of *cup-14(cd32)* worms identified homozygous, protein-altering mutations in two genes on chromosome II, *Y51H1A.2 and sre-46*. RNAi of *Y51H1A.2*, but not of *sre-46*, yielded the *cup-14* mutant bright coelomocyte GFP defect in P*myo-3*::*ssGFP* worms. Furthermore, *cup-14(cd32)*; P*myo-3*::*ssGFP* did not complement an existing null allele of *Y51H1A.2* (Table S1). Indeed, a *Y51H1A.2(gk595958)*; P*myo-3*::*ssGFP* strain showed the same Cup phenotype as *cup-14*; P*myo-3*::*ssGFP*. We identified homozygous mutations in *Y51H1A.2* in both *cup-14* alleles; *cd31* is a missense allele, and *cd32* creates a stop codon that truncates the carboxyl terminus (Figure S3). Thus, CUP-14 is Y51H1A.2, which is homologous to the endolysosomal PLEKHM1 protein in mammals (McEwan *et al.* 2015a,b).

#### cup-15:

We isolated two independent alleles of *cup-15* (Table 1). We mapped *cup-15(cd33)* to chromosome II. Whole-genome sequence analysis of *cup-15(cd33)* worms identified a homozygous, nonsense mutation in *F42A8.3*, and homozygous, missense mutations in *bmy-1*, *cpna-2*, *T01H3.2*, *efl-3*, and *F14E5.1* on chromosome II. *cup-15(cd33)*; P*myo-3*::*ssGFP* complemented existing null alleles of *cpna-2* and *T01H3.2*, but did not complement an existing null allele of *F42A8.3* (Table S1). Indeed, an *F42A8.3(gk150714)*; P*myo-3*::*ssGFP* strain showed the same Cup phenotype as *cup-15*; P*myo-3*::*ssGFP*. Furthermore, RNAi of *F42A8.3* yielded the *cup-15* mutant bright coelomocyte GFP defect in P*myo-3*::*ssGFP* worms. We confirmed the presence of homozygous mutations in *F42A8.3* in both *cup-15* alleles; *cd33* is a nonsense mutation that truncates the carboxyl terminus, including a transmembrane domain, and *cd46* is a deletion that removes from upstream of the predicted start codon to the middle of the second intron (Figure S4). Thus, CUP-15 is F42A8.3, which is homologous to mammalian OSTM1 that forms the Cl^−^/H^+^ exchange channel with mammalian CIC-7 (worm CLH-6/CUP-12) on lysosomes (Lange *et al.* 2006; Leisle *et al.* 2011).

#### cup-16:

We isolated one allele of *cup-16* (Table 1). We mapped *cup-16(cd50)* to chromosome II. Whole-genome sequence analysis of *cup-16(cd50)* worms identified homozygous, protein-altering mutations in several genes on chromosome II: *cpb-2*, *srh-60*, *W10G11.17*, F19B10.1, *ani-2*, *F18A1.1*, *cpna-2*, *C34C6.7*, *cct-2*, *C18E9.9*, *T15H9.6*, *and let-23. cup-16(cd50)*; P*myo-3*::*ssGFP* did not complement an existing null allele of *C34C6.7*, but complemented missense (*srh-60*, *W10G11.17*, *C18E9.9*) and nonsense (*cpna-2*, F19B10.1, *ani-2*, *F18A1.1*, *C34C6.7*, *cct-2*, *T15H9.6*, *let-23*) alleles in the other genes (Table S1). Indeed, a *C34C6.7(gk358192)*; P*myo-3*::*ssGFP* strain showed the same Cup phenotypes as *cup-16*; P*myo-3*::*ssGFP*. Furthermore, RNAi of *C34C6.7* yielded the *cup-16* mutant bright coelomocyte GFP and body cavity GFP defects in P*myo-3*::*ssGFP* worms. *cd50* is a missense mutation (Figure S5). Thus, CUP-16 is C34C6.7, which, based on primary sequence blast searches, appears to be found only in the *Caenorhabditis* genus.

#### cup-17:

We isolated one allele of *cup-17* (Table 1). We mapped *cup-17(cd49)* to chromosome II. Whole-genome sequence analysis of *cup-17(cd49)* worms identified homozygous, nonsense mutations in *C41C4.7/ctns-1* and *T19H5.2/chil-26*, and missense mutations in *ZK1127.4*, *ZK1290.13*, *cec-3*, *C18E9.7*, *T24H10.1*, *R09D1.4*, *mrps-22*, *kin-6*, *F13D12.8*, and *F44E5.3* on chromosome II. *cup-17(cd49)*; P*myo-3*::*ssGFP* did not complement an existing null allele of *ctns-1* (Table S1). Indeed, a *ctns-1(ok813)*; P*myo-3*::*ssGFP* strain showed the same Cup phenotype as *cup-17*; P*myo-3*::*ssGFP*. Furthermore, RNAi of *ctns-1* yielded the *cup-17* mutant bright coelomocyte GFP defect in P*myo-3*::*ssGFP* worms. *cd49* is a nonsense mutation that truncates the carboxyl terminus, including a transmembrane domain (Figure S6). Thus, CUP-17 is CTNS-1, which is homologous to the lysosomal cysteine transporter Cystinosin in mammals, and to *S. cerevisiae* ERS1 (Kalatzis *et al.* 2001; Gao *et al.* 2004; Liu *et al.* 2012).

Having identified the genes from our mutational screen, we analyzed endolysosomal transport to identify which aspects of lysosome biology they regulate.

### Determining organelle sizes to evaluate membrane traffic

The sizes of endosomes and lysosomes increase with membrane that they receive from other compartments through fusion reactions, and decrease with membrane that they lose through fission reactions (for example, vesicular or tubular transport). To determine where the CUP proteins functioned in the endocytic pathway, we used established markers for organelles to evaluate sizes of endosomes and of lysosomes. We included the *cup-5* mutant in this analysis for comparative purposes.

**Figure 2:**
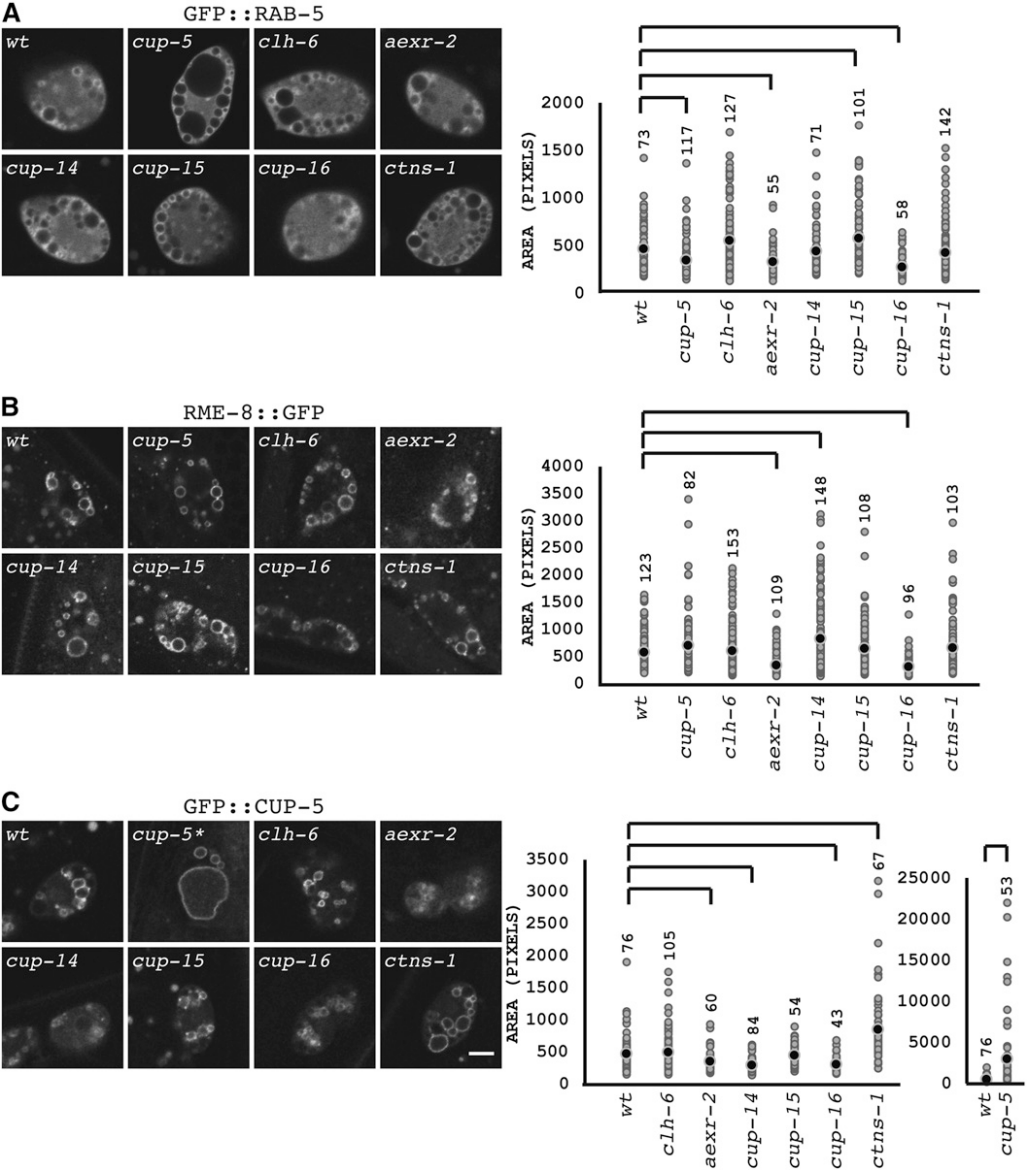
Sizes of endolysosomal compartments. (A) Confocal images of coelomocytes in young adult hermaphrodites of the indicated genotypes that also carry the *cdIs131[*P*cc1*::*GFP*::*rab-5*; *unc-119-myo-2*::*GFP]* transgene. The scatter plot shows the sizes of individual GFP::RAB-5-labeled compartments in the coelomocytes. (B) Confocal images of coelomocytes in young adult hermaphrodites of the indicated genotypes that also carry the *cdIs153[rme-8*::*GFP*; *unc-119-myo-2*::*GFP]* transgene. The scatter plot shows the sizes of individual RME-8::GFP-labeled compartments in the coelomocytes. (C) Confocal images of coelomocytes in young adult hermaphrodites of the indicated genotypes that also carry the *cdIs40[*P*cc1*::*GFP*::*cup-5*; *unc-119-myo-2*::*GFP]* transgene; the *cup-5* mutant carries the *pwIs50[lmp-1*::*GFP*; *unc-119]* transgene. The left scatter plot shows the sizes of individual GFP::CUP-5-labeled compartments in the coelomocytes; the *cup-5* mutant is shown on a separate chart to adjust for scale. The black circle indicates the average size of organelles. The number above each column indicates the number of organelles measured for each strain. Inverted brackets indicate *P* < 0.05 of mutants compared to wild type. Bar = 5 µm.

We first used GFP::RAB-5 as a marker of early endosomes (Dang *et al.* 2004; Patton *et al.* 2005; Schaheen *et al.* 2009). The *cup-5* (*ar465*) mutant showed early endosomes that were 70% the size of early endosomes in wild-type coelomocytes (*P* 4.3 × 10^−5^); however, the large vacuoles that push against other compartments in the *cup-5* mutant make determination of early endosome sizes inaccurate (Figure 2, A and C). The *cup-15* mutant showed slightly larger early endosomes than wild-type cells (1.24-times larger; *P* 0.02), suggesting increased endocytic uptake, increased early endosome fusion, or decreased membrane transport from early endosomes in the absence of CUP-15 (Figure 2A). The most significant differences were decreased early endosome sizes compared to wild-type coelomocytes in the *cup-13/aexr-2* mutant (70% of wild type; *P* 2.6 × 10^−4^) and the *cup-16* mutant (58% of wild type; *P* 1.5 × 10^−8^); these are consistent with the decreased endocytic uptake of GFP by coelomocytes, and the accumulation of GFP in the body cavities of *cup-13/aexr-2* and *cup-16* mutants (Figure 1B and Figure 2A).

Proceeding along the endolysosomal pathway, we next used RME-8::GFP as a marker of early and late endosomes (Zhang *et al.* 2001; Treusch *et al.* 2004; Patton *et al.* 2005). Both the *cup-13/aexr-2* mutant (58% of wild type; *P* 2.3 × 10^−9^), and the *cup-16* mutant (52% of wild type; *P* 10^−11^), showed decreased sizes consistent with the decreased amount of membrane transported to endosomes and lysosomes in these mutants (Figure 2B). In addition, the *cup-14* mutant showed slightly larger early/late endosomes than wild-type cells (1.4-times larger; *P* 1.5 × 10^−4^); the normal sized GFP::RAB-5-marked early endosomes in the *cup-14* mutant suggest a trafficking defect from late endosomes in the absence of CUP-14 (Figure 2B).

We next analyzed lysosomes using a GFP::CUP-5 marker for these organelles; for the *cup-5* mutant, we used an LMP-1::GFP fusion to assay the sizes of the terminal compartments, since GFP::CUP-5 rescues the *cup-5* mutation (Treusch *et al.* 2004). Consistent with the reduced sizes of early and late endosomes, both the *cup-13/aexr-2* mutant (74% of wild type; *P* 3.5 × 10^−3^) and the *cup-16* mutant (52% of wild type; *P* 1.9 × 10^−4^) showed decreased sizes of lysosomes (Figure 2C). In addition, the *cup-14* mutant showed severely reduced lysosome sizes relative to wild type (61% of wild type; *P* 7.4 × 10^−8^), suggestive of a defect in lysosome homotypic fusion or increased homotypic lysosome fission (Figure 2C). The *cup-17/ctns-1* mutant showed significantly increased sizes of terminal compartments compared to wild type (1.8 times larger, *P* 3.6 × 10^−7^), though that increase was much more pronounced in the *cup-5* mutant that showed terminal compartments that were, on average, 6.4 times larger than wild type (Figure 2C).

The analysis of endolysosomal compartment sizes revealed significant defects in the *cup-13/aexr-2*, *cup-14*, *cup-16*, and *cup-17/ctns-1* mutants. Given the defect in GFP degradation in all of the mutants, this suggested that some mutants had milder transport defects that caused a delay in the degradation of endocytosed GFP without grossly affecting sizes of late endosomes and lysosomes, at least at this resolution. We therefore followed the membrane transport of an endocytosed substrate to assay the efficiency of trafficking to lysosomes in the *cup* mutants.

### Pulse-chase analysis of endocytosed substrate

In P*myo-3*::*ssGFP* worms, the GFP that is endocytosed by coelomocytes from the body cavity is visible primarily in lysosomes, where it accumulates and is eventually degraded (Fares and Greenwald 2001a; Treusch *et al.* 2004). We used pulse-chase analysis of a fluorescent substrate injected into the body cavities of worms to visualize the rate of flow of endocytosed substrate through endosomal compartments to lysosomes in coelomocytes. BSA-Alexa Fluor 594 microinjected into the body cavity appears by 5 min in endosomal compartments, and is concentrated in small nascent lysosomes budding from hybrid organelles; by 30 min the BSA-Alexa Fluor 594 is fully localized to lysosomes (Figure S7) (Zhang *et al.* 2001; Dang *et al.* 2004; Treusch *et al.* 2004; Schaheen *et al.* 2009).

**Figure 3:**
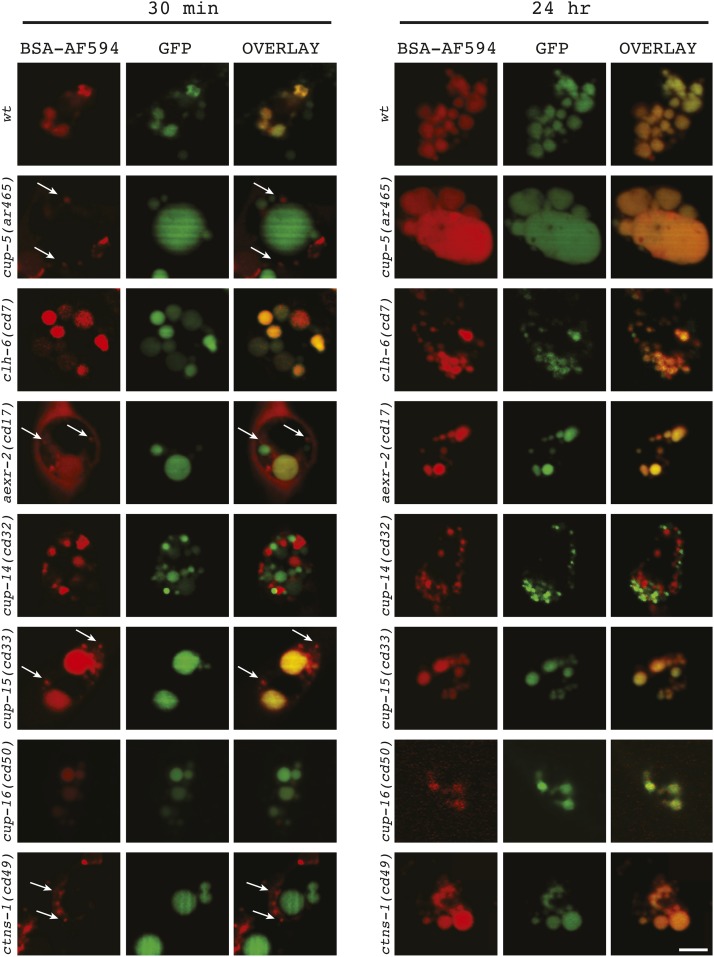
Endolysosomal transport of endocytosed BSA-Alexa Fluor 594. Single optical section confocal images of the indicated genotypes also carrying the *arIs37[*P*myo-3*::*ssGFP*; *dpy-20]* transgene at the indicated times after microinjection of BSA-Alexa Fluor 594 into their body cavities. Arrows indicate nascent lysosomes containing concentrated BSA-Alexa Fluor 594. Bar = 5 µm.

We microinjected BSA-Alexa Fluor 594 into the body cavity of P*myo-3*::*ssGFP* worms and imaged two time points, 30 min and 24 hr; at these time points, the BSA-Alexa Fluor 594 is completely found in lysosomes of wild type worms, where it colocalizes with endocytosed GFP (Figure 3 and Figure S7). Pulse-chase analysis with microinjected BSA-Alexa Fluor 594 into the body cavities of the mutants revealed three types of behavior:

1. *cup-12/clh-6* and *cup-16* mutants showed normal trafficking of BSA-Alexa Fluor 594, such that all of the BSA-Alexa Fluor 594 was colocalized with GFP-filled compartments by 30 min (Figure 3). Thus, there is no delay in the transport of endocytosed BSA-Alexa Fluor 594 to lysosomes in *cup-12/clh-6* and *cup-16* mutants.
2. Similar to *cup-5* mutants, *cup-13/aexr-2*, *cup-15*, and *cup-17/ctns-1* mutants showed a delay in the transport of BSA-Alexa Fluor 594 to lysosomes, such that there were still nascent lysosomes with concentrated BSA-Alexa Fluor 594 after 30 min; the BSA-Alexa Fluor 594 was fully localized to GFP-filled compartments by 24 hr (Figure 3) (Treusch *et al.* 2004). Between these three mutants, the *cup-17/ctns-1* mutant had the most severe defect in transport to lysosomes, such that no BSA-Alexa Fluor 594 had reached GFP-filled lysosomes by 30 min; in contrast, *cup-13*/aexr-2 and *cup-15* mutants had a milder defect, such that some BSA-Alexa Fluor 594 had reached GFP-filled compartments by 30 min (Figure 3). Thus, CUP-13, CUP-15, and CUP-17 likely function in the process of lysosome formation, that is, the efficient scission of nascent lysosomes budding from late endosomes/hybrid organelles.
3. The *cup-14* mutant did not show a delay in transport to lysosomes since there were no BSA-Alexa Fluor 594 concentrations in nascent lysosomes by 30 min (Figure 3); however, the BSA-Alexa Fluor did not colocalize with GFP-filled lysosomes after 30 min, or even after 24 hr (Figure 3). This implicates CUP-14 in the fusion of lysosomes with each other (see *Discussion*).

Thus, the mutants we identified affect at least four different aspects of lysosome biology.

## Discussion

Lysosomes are one of the major degradative organelles in eukaryotic cells that carry out diverse cellular functions. Lysosomes show highly dynamic behaviors, including homotypic and heterotypic fusions, fission, and formation/reformation, which itself involves budding, extension, and scission. We carried out an unbiased forward mutational screen to identify novel regulators of lysosome dynamics and/or function; this screen is based on the degradation of a substrate, GFP, that is endocytosed by scavenger cells in worms. We identified *cup-5* and six additional proteins that have lysosomal functions in *C. elegans* coelomocytes. CUP-16 is only conserved in the genus *Caenorhabditis*, and likely functions in endocytic uptake at the plasma membrane and in lysosomal degradation. Besides CUP-16, five of the mammalian homologs of the other CUP proteins, CIC-7, OSTM1, PLEKHM1, Cystinosin, and TRPML1, had been previously implicated in lysosome biology, thus validating this approach (Bach 2001; Lange *et al.* 2006; Wartosch *et al.* 2009; Weinert *et al.* 2010; Leisle *et al.* 2011; Ivanova *et al.* 2015; McEwan *et al.* 2015a,b; Andrzejewska *et al.* 2016). There are certainly additional genes that function in lysosome biology, some of which are essential for embryonic development, and would require isolating rarer hypomorphic mutations that impair lysosomal function without affecting viability, as was the case for *cup-5*. In addition, we relied on a visual screen of whole worms, so more subtle defects in lysosome function or structure would have been missed. However, we have identified regulators and generated novel insights into at least four different steps in the endocytic pathway (Figure S8).

First, mutation of the G protein-coupled receptor CUP-13/AEXR-2 results in an early defect in endocytic uptake of a solute from the extracellular space. G protein-coupled receptors are known to function in signaling pathways at the plasma membrane through their coupling to heterotrimeric G proteins. The activities of G protein-coupled receptors is regulated by their internalization into early endosomes, followed by their recycling to the plasma membrane or their degradation in lysosomes (Seachrist and Ferguson 2003; Tsvetanova *et al.* 2015). Our study shows that, besides regulating their own transport and activities, G protein-coupled receptors can regulate general endocytic transport.

Second, we identified CUP-13/AEXR-2, CUP-15, and CUP-17/CTNS-1 as novel regulators of the poorly understood process of lysosome formation (Figure S8). This is based primarily on an apparently normal endocytic uptake of BSA-Alexa Fluor 594 in the mutants, and the persistence of nascent lysosomes with concentrated BSA-Alexa Fluor 594 at later time points of the pulse-chase analysis; however, this does not preclude additional roles of these proteins in other lysosomal trafficking events, for example, fusion of late endosomes with lysosomes. Based on the mutant phenotypes in *C. elegans*, and our quantitative live imaging studies in mammalian cells, CUP-5/TRPML1 plays a central role in lysosome formation (Treusch *et al.* 2004; Miller *et al.* 2015). The mutant phenotypes suggest that, though the activities of CUP-13/AEXR-2, CUP-15, and CUP-17/CTNS-1 may not be strictly essential for the completion of the budding, extension, and scission events during lysosome formation, these proteins modulate the efficiency of lysosome formation. It is currently unclear how G protein-coupled receptors (CUP-13/AEXR-2), OSTM1 (CUP-15), and Cystinosin (CUP-17/CTNS-1) regulate lysosome formation, and whether the defects in this process in *C. elegans* mutants are an indirect consequence of their reduced or absent function. OSTM1 and Cystinosin have roles in ion and cysteine transport across membranes, respectively, such that the lysosome formation defect in the *cup-15* and *cup-17/ctns-1* mutants may be an indirect consequence of altered ion/cysteine homeostasis (Kalatzis *et al.* 2001; Lange *et al.* 2006; Leisle *et al.* 2011; Liu *et al.* 2012). Alternatively, these proteins may have direct roles in lysosome formation, either dependent or independent of their channel functions, for example, Cystinosin has dual and independent roles as a cysteine transporter, and as a component of the mTORC1 pathway (Andrzejewska *et al.* 2016; Ivanova *et al.* 2016).

Third, our studies indicate a role for CUP-14 in promoting primary lysosomes fusing with each other (Figure S8). In the absence of CUP-14, endocytosed GFP and BSA-Alexa Fluor 594 localize to discrete lysosomes that are reduced in size. This is in agreement with our identification of scavenger receptors that are required for the endocytosis and for the transport from late endosomes to lysosomes of endocytosed GFP, but not of BSA (H.F. unpublished data). Our model is that scavenger receptors localize to nascent lysosomes where they concentrate certain solutes (like GFP or BSA). Following scission, CUP-14 and other proteins, yet to be identified, regulate the fusion of these primary lysosomes, resulting in content mixing (Figure S8). Our results further suggest that primary lysosomes are not all identical, or “homotypic”; primary lysosomes that carry GFP may be homotypic, primary lysosomes that carry BSA may also be homotypic, but the GFP-bearing lysosomes and the BSA-bearing lysosomes likely have different scavenger receptors and possibly other constituents, and may thus require a specific machinery, that includes CUP-14, for their fusion with each other. PLEKHM1, the mammalian homolog of CUP-14, functions in “heterotypic” lysosomal fusion events with *Salmonella*-containing vacuoles and autophagososmes (McEwan *et al.* 2015a,b). Our study extends PLEKHM1 functions to other lysosomal fusion events, and supports a model of receptor-mediated concentration of solutes in nascent lysosomes. CUP-14 may also have earlier roles in late endosomes, as evidenced by the slightly larger RME-8-labeled compartments in the *cup-14* mutant. These larger late endosomes are not due to a defect in membrane transport to lysosomes, as determined by the BSA-Alexa Fluor 594 pulse-chase analysis. One possibility is that the lysosome fusion defect in the absence of CUP-14 makes these smaller lysosomes more fusogenic with late endosomes, thus increasing the sizes of the large endosomes; alternatively, the *cup-14* mutant may have defects in membrane transport from late endosomes to other cellular organelles besides lysosomes, for example the Golgi apparatus.

Fourth, mutation of CUP-12/CLH-6 does not affect membrane transport steps, but likely reduces lysosomal degradative functions (Figure S8). This is in agreement with studies on the mammalian homolog CIC-7, which functions as a Cl^−^/H^+^ exchange protein that is required for lysosome acidification (Wartosch *et al.* 2009; Weinert *et al.* 2010). Mammalian studies identified OSTM1 (CUP-15) as a beta subunit of the CIC-7 channel that is required for the lysosomal transport and the channel activity of CIC-7 (Lange *et al.* 2006; Wartosch *et al.* 2009; Leisle *et al.* 2011). Our studies suggest additional functions for OSTM1 (CUP-15) in the regulation of lysosome formation that are independent of its roles with CIC-7 (CUP-12/CLH-6).

CIC-7, OSTM1, Cystinosin, and TRPML1 are all associated with human diseases (Bach 2001; Kalatzis *et al.* 2001; Lange *et al.* 2006; Weinert *et al.* 2010). Our study has identified potentially novel roles of these and other proteins in lysosome biology; this allows more accurate investigations into the basis for lysosomal defects in human disorders.

## Supplementary Material

Supplemental material is available online at www.g3journal.org/lookup/suppl/doi:10.1534/g3.116.037515/-/DC1.
