# Supplementary material for: Regulators of Lysosome Function and Dynamics in Caenorhabditis elegans
Source: G3 (Bethesda). 2017 Jan 24;7(3):991–1000. doi: 10.1534/g3.116.037515 (PMC5345728; doi:10.1534/g3.116.037515)
Supplement: Supplementary file 1 [file 991FigureS1.docx]

***clh-6* predicted open reading frame ATG-STOP**

ATG ACA TCC CGT AAC AGG GTG CGA ACA AGT TCT AAT AGT CTG GAT CCA AAT GAA GAT CCT GTG TCG GGG CTA GAA CGA GTC ATG TCA AAC TCG AAT CTA GAA 102

M T S R N R V R T S S N S L D P N E D P V S G L E R V M S N S N L E 34

CAC GTT GCT CGG CAA AGA AGA CGA CTA GAG GAA GAG CTG TTA TTC TCA AGG CAA TTG GAT GAT GTT GGT CGA ACG GAT AGC CAT GAA GCA TTG TCT GCT AGA 204

H V A R Q R R R L E E E L L F S R Q L D D V G R T D S H E A L S A R 68

TAC GAA AGT CTG AAC TAT GAA ATA TCT GAG AAT CGA CTC TAT AGA GAT GCT GAA AAG AAA CCA TCT CAC CAG TTA ACT CTT TGG CGA ATT TCT CGA AAT CGT 306

Y E S L N Y E I S E N R L Y R D A E K K P S H Q L T L W R I S R N R 102

TGG TTT GTA TGC TTT TTG ATT GGA GTT TTC ACT GGA CTC GTC GCT GCA TTT ATT GAT ATT ATG GTA CAC TAC AGT AAA GAT ATC AAA TTT AAC TGG ATT CTC 408

W F V C F L I G V F T G L V A A F I D I M V H Y S K D I K F N W I L 136

AAA TAT TTG CTC TCA AAG TGT GGC GAA GAG CAA CGT GGA ACG ACT GCA GGG TGT ATG TGG ACC GTA ATG ATA GCT TGG ATA GGA TAT AAT TGT GTT CTC GTA 510

K Y L L S K C G E E Q R G T T A G C M W T V M I A W I G Y N C V L V 170

ACT ATA GCT GCA ATC CTT GTG ATT TAT GTA GCT CCG ATT GCT GGA GGT TCT GGA ATT CCT CAA ATA AAG TGT TAT CTT AAC GGA ATT GCG ATT CCT GAG GTT 612

T I A A I L V I Y V A P I A G G S G I P Q I K C Y L N G I A I P E V 204

GTT CGT CTC AAA ACG CTC GTT TCA AAA GCA GTT GGA GTT GCA TGT TCG GTT GGC GGT GGT CTT TGT GCT GGA AAA GAA GGT CCT ATG ATC CAT TCT GGA GCT 714

V R L K T L V S K A V G V A C S V G G G L C A G K E G P M I H S G A 238

GCT GTA GGA GCT GGG ATA TCA CAG GGA AAA AGT TAT TCC CTT GGA ATC GAT TTC GGA CTT TTC CGC GAG TTT AGA AAT GAT CGA GAA AAA AGA GAT TTC GTT 816

A V G A G I S Q G K S Y S L G I D F G L F R E F R N D R E K R D F V 272

T in *cd14*

TCT GCT GGT GCT GCT GCT GGA GTT GCT GCT GCT TTC GGA GCA CCA ATC GGA GGA GTT CTA TTC TCT TTA GAA GAA GGA GCA AGC TTT TGG AAT CAA GCT TTG 918

S A G A A A G V A A A F G A P I G G V L F S L E E G A S F W N Q A L 306

V in *cd14*

ACA TGG AGA ATG TTT TTC TCT GCA ATG ATC TCT TCA TTC ACT GTA AAT TGG ATT CTC AGT TGG TTC AAT GGA CGT AGT GGA TGG CTC TCA TGG ACA GGT CTC 1020

T W R M F F S A M I S S F T V N W I L S W F N G R S G W L S W T G L 340

GCG AAT TTC GGA GTT TTC GAA AAT AAG GAT TAT AAT ATT TGG GAA ATT CCT TTG TTT CTT CTC ATT GGA ATA ATT GGA GGC TGT CTT GGA GCT TTA TTC AAT 1122

A N F G V F E N K D Y N I W E I P L F L L I G I I G G C L G A L F N 374

T in *cd34*

TAT TTA AAT ACA AAA CTC ACC GAG TTC CGT AAA AAA TAT GTG AGC AGC AAA CTG GGT CGT TTG TTC GAA TGT CTT CTT GTT GCC GCG GTA TCA GGA TTT CTT 1224

Y L N T K L T E F R K K Y V S S K L G R L F E C L L V A A V S G F L 408

C in *cd34*

GCT TTC CTC ACA ATA TTC GCA ATT GAC GAT TGT CAG CCG ATA GGA GCG AAT CCT ACA GCA ACA TCA ACA CAA ATC AAT CAA ATG TGG TGC AAA AAA GGA GAA 1326

A F L T I F A I D D C Q P I G A N P T A T S T Q I N Q M W C K K G E 442

TAT TCT GCA GTA GCT TCT TTA TTT TTT CAA AAT CCT GAA GAA AGT GTG AAG AGT ATG TTT CAT AGT CCG ATA AAT TCT TTT GGT GTC ACA ACT CTT GTT ATT 1428

Y S A V A S L F F Q N P E E S V K S M F H S P I N S F G V T T L V I 476

TTT GGA ATT GAA TAT TTC TTG CTC ACT CTC TGG ACG TTC GGA ATC TCT GTA CCT TCT GGT GTC TTC ATT CCA GCT ATT CTT ACT GGT GCA GCA TGG GGC CGC 1530

F G I E Y F L L T L W T F G I S V P S G V F I P A I L T G A A W G R 510

A in *cd7*

CTG TTT GGA ATT TTT GTT GAA CGA CTT TTC CCT TCG GTT ACT GGA ATT GAT CCA GGA AAA TAC GCA TTG GCA GGA GCT GCC GCA CAA CTC GGA GGC GTT GTT 1632

L F G I F V E R L F P S V T G I D P G K Y A L A G A A A Q L G G V V 544

E in *cd7*

T in *cd39*

CGA ATG ACA ATT TCA TTA ACA GCT ATT ATA ATG GAA GCA ACT AAA GAT ATT ACA TTT GGA TTA CCT ATT ATG TTG GTG TTG ATG GTA ACA AAA TGG GTT GGA 1734

R M T I S L T A I I M E A T K D I T F G L P I M L V L M V T K W V G 578

F in *cd39*

A in *cd15*

GAT ATG TTC AAT GAA GGA CTT TAT GAT GCT CAT ATT GAT TTA GCA GAA GTT CCA ATT CTT GGT TGG AAT CCA CCA AAG ATG AGC AGA AAC ATT TTG GCA GAC 1836

D M F N E G L Y D A H I D L A E V P I L G W N P P K M S R N I L A D 612

* in *cd15*

CGC GTG ATG CGA AAA GAT GTT GTG GCT CTA GAA CGG CGA GAA AGA GTA TCC AGA ATC GTT GAA ATT CTT CGT TCC ACA CTT CAT CAT GGC TTT CCA GTA GTA 1938

R V M R K D V V A L E R R E R V S R I V E I L R S T L H H G F P V V 646

GAT AGA ATC GAA GAA TCG CCA TAC GAA TCA CTC CCT GAT TAT GGA CGC CTG AAA GGT TAT ATT CTG CGA TCA CAA CTG TTC AAA CTT CTG GAA AAT CGA ATT 2040

D R I E E S P Y E S L P D Y G R L K G Y I L R S Q L F K L L E N R I 680

TTC GAG GAA GAA GGA GCT TCA AAC TCT GCA CTT CCA AAT GAT TTT TAT GAA TGC CAG GAT GAC GAC GAC CAA ATG AAA TCT GTT GCT GAA CTC GGA TTA ACA 2142

F E E E G A S N S A L P N D F Y E C Q D D D D Q M K S V A E L G L T 714

AAT TTC GAC GAG TCA TGT TTC CTG GAC ATT GAG CCC TAC ATG CAT CCT CAT CCG CAC CGT GTT CCA CTG AAC ACA TCT CTT CCA TTC ATT TTC CGA CTT TTC 2244

N F D E S C F L D I E P Y M H P H P H R V P L N T S L P F I F R L F 748

CGT GGA CTT GGA CTT CGA TAC CTT TTT GTG GTG AAC GAT GAT AAT CAT CTT CGT GGT GTG ATT ACT AGA AAA GAT GTT GCC AGA TTC CGT GAA AGA CGC CGA 2346

R G L G L R Y L F V V N D D N H L R G V I T R K D V A R F R E R R R 782

AAC CGC GAG TAT CAT GTT GAT GAA CTT TAT ATT TCT GAA TCT TAA 2391

N R E Y H V D E L Y I S E S * 796

**Figure S1** Predicted Open Reading Frame of *cup-12/clh-6*. The transmembrane domains are highlighted in yellow; the CBS domains are highlighted in green. Changes to the DNA and the protein sequences in *cup-12* alleles are indicated.
