# Supplementary material for: Regulators of Lysosome Function and Dynamics in Caenorhabditis elegans
Source: G3 (Bethesda). 2017 Jan 24;7(3):991–1000. doi: 10.1534/g3.116.037515 (PMC5345728; doi:10.1534/g3.116.037515)
Supplement: Supplementary file 2 [file 991FigureS2.docx]

***aexr-2* predicted open reading frame ATG-STOP**

ATG GCA TTT TCA TTG AGC CAA CGA GTC GTG GAA ACG ATG CCA TCT GGA ACA TGG AAC GAT TTC ACT GAT CTT CCG GCA GAA TTC ATT TCT CAA TTT GGA TCT 102

M A F S L S Q R V V E T M P S G T W N D F T D L P A E F I S Q F G S 34

TCT TCC GAG GCA ATG CAG TAT TCA TGT TAT TAC TCC GAT TCC TTT ATT CGC TTT GCA ACC GCT ATG GAC GAC GTA TTA ATT GGA GCA TGT CTC ATA TCT ACT 204

S S E A M Q Y S C Y Y S D S F I R F A T A M D D V L I G A C L I S T 68

GTG ATT AAT TTT ATC GTG ATT GCA TGT TCC GCC AAG TTA TAT AAA AAG AAA GGA GAC ACA CTT CAC TTG TTT ATT TTG AAC ATG ACT ATT GGA GAC ACA ATA 306

V I N F I V I A C S A K L Y K K K G D T L H L F I L N M T I G D T I 102

A in *ar482*

TTA ACA CTG TTT TGC CAT CCG TAT GAG CTA GTC ACG AGA AGA TAT TCT GGA GCA CAT GTT CAT TTC ATA ACT GTT TTT TTG AAT TTT GCA AAC TGG GTG GGT 408

L T L F C H P Y E L V T R R Y S G A H V H F I T V F L N F A N W V G 136

D in *ar482*

A in *cd16* A in *ar487*

CTT GCA GTA TCC GGG CTT TCA CTC ACT TTG CTG AAT ATT GAT AAG CTA ATA TTT TTT TGC TGG CCA TTC AAA TAT GAC ATA TGG ATG TCT TAT TTT AGA GCC 510

L A V S G L S L T L L N I D K L I F F C W P F K Y D I W M S Y F R A 170

T in *cd16* E in *ar487*

A in *cd17* A in *ar472* and *ar480*

AAG CTA TTT TGT TAT CTC TCC TGG ATT ATA TCA ATA GGT TTT GCG ACA TAC TAC TGG ATG TAT AGT TAT ATG TAC TTT GTA AAT GCA ACA GTT GAT ATT CAA 612

K L F C Y L S W I I S I G F A T Y Y W M Y S Y M Y F V N A T V D I Q 204

D in *cd17* * in *ar472* and *ar480*

T in *ar510*

TTT TCT CCG GTG AAC AAA ATT TTC TAC GAA GTA TTC ACA GTG GTG TTC TGT GTT ATT CCG ATA GTG TCA TCG CTG TTA GTA TCC TGT TAC TTA TAC GAT TTA 714

F S P V N K I F Y E V F T V V F C V I P I V S S L L V S C Y L Y D L 238

S in *ar510*

A in *ar503*

ACT AAA AGG AAA CGA AAA ACC GTG ATT AAG ACT AGC AGT GCA AGC AAA ATT GAG AAT AAA GCA ACA TCT TTT GCA TTC ATC TTC GCG ACA ACA CTT TGG ACA 816

T K R K R K T V I K T S S A S K I E N K A T S F A F I F A T T L W T 272

* in *ar503*

A in *ar491*

TCT TGC AGT TTG CTT CCT TAT AGA ATA GCG AAT CTA GCG AGA ATT CAT ATT ATA GCG TGG CCC AAC CTA GAC TGT GAA TCT CGT CAA AAC TTG AGC TGG CTG 918

S C S L L P Y R I A N L A R I H I I A W P N L D C E S R Q N L S W L 306

* in *ar491*

T in *ar501*

ACC TGG AGT ATG CTT TAC TTA CTT ATT TTG AAT CCA ATA ATT AAT CCC CTC ATA ACT GCA TTT GCC TAC GCG CCA TAC CGT CAG ATG ATA TAC TCA AGA GTT 1020

T W S M L Y L L I L N P I I N P L I T A F A Y A P Y R Q M I Y S R V 340

L in *ar501*

CGG AAA TCA TCT AGA AAA AAC TAT CAA GAT CAG AAT TCC TAC GAA ACA AAC AAA AGC AAC ATA ACA TCA ACA AAT TCT GTA TCC TCG AAA GTG TTT TAT GTT 1122

R K S S R K N Y Q D Q N S Y E T N K S N I T S T N S V S S K V F Y V 374

GAT CTC ACG ATG GCT AAA CAG AAC CAT ACC AAA ACT TTG AGG AAA GCT CCA CTT GCC AAG TTC CCA AGC ATT TGC TCC TGC GGC TCT TTT GTG ACC AAC GAA 1224

D L T M A K Q N H T K T L R K A P L A K F P S I C S C G S F V T N E 408

AAT CTA CAG TGC ACA CGG TTT TAG 1248

N L Q C T R F * 415

**Figure S2** Predicted Open Reading Frame of *cup-11/cup-13/aexr-2*. The transmembrane domains are highlighted in yellow. Changes to the DNA and the protein sequences in *cup-11* and *cup-13* alleles are indicated.
