# Supplementary material for: Regulators of Lysosome Function and Dynamics in Caenorhabditis elegans
Source: G3 (Bethesda). 2017 Jan 24;7(3):991–1000. doi: 10.1534/g3.116.037515 (PMC5345728; doi:10.1534/g3.116.037515)
Supplement: Supplementary file 3 [file 991FigureS3.docx]

***Y51H1A.2a* predicted open reading frame ATG-STOP**

ATG TTC AAA CGG CTG AAT AAA ACC ACC CAA ATG GCC TCA GCG CCC CCC CAA TTA ACA CCG GCT CAG AAA CTG AAA CAG GAG AAA ATC CGT GCG GAT TTG CAA 102

M F K R L N K T T Q M A S A P P Q L T P A Q K L K Q E K I R A D L Q 34

AAG GAG CTT GAT AAT GTT GTG AAG GCT GCT ATA GCT TCT CCG CAC TCG AAA AGG GAG AAT GTG CCG AGC GAA ATC ACC CAA AAT CTG TGC AAT TCG ATC GAA 204

K E L D N V V K A A I A S P H S K R E N V P S E I T Q N L C N S I E 68

GCA ATC TTC ATT CAC GGA CTA CGA GAC CCA TTT TTC TTA AAA GGA ACA AGA TAT GCC AAA TAT CCA GAG CCA AAT TTC TGG CCA TTT GTC TCG AAA TTC TCG 306

A I F I H G L R D P F F L K G T R Y A K Y P E P N F W P F V S K F S 102

CAT CGA TCG ATA ACC AGC CAA ATC GCT TGC CTC GGA CAA ATT CGG AGT GAA ATT GGA AAA AGC CGG GCC TGG ATT CGA ATT GTG TTG AAT GAA AAT GCA CTG 408

H R S I T S Q I A C L G Q I R S E I G K S R A W I R I V L N E N A L 136

T in *cd31*

GGA CAG TAT TTA GAT CTA CTG GCC GCT GAG GCG ACT GCT ATT CAA CAA TTC TAC TCT GAC GAC GCA TTT CTC CGG CTT CTC TCC GAT GGA GAT CAA TCA GAA 510

G Q Y L D L L A A E A T A I Q Q F Y S D D A F L R L L S D G D Q S E 170

V in *cd31*

CGA ATC CGT GGC CTA CTA AAA CCC CTC AGC TCA CTT CCA ATA TCT GCA GCA ACT AAT TCA TCA TTT CTT AAC ACT TGG ACA CCA ACT CCA TTA ATT TTA GCC 612

R I R G L L K P L S S L P I S A A T N S S F L N T W T P T P L I L A 204

GGT CTA ATG GAT GGT CAA CCA CTA AAA GTT GGC ACT TTA AAA GCT CGT CCC AAC CCG AAA CCG GCT CAT TTA ACA GAG GAA ATT GCG ATT CCG GCG ATT GAT 714

G L M D G Q P L K V G T L K A R P N P K P A H L T E E I A I P A I D 238

GCT CTT GTG CCG GAG GAA GAT CAT GAT ATT GGA TCA CCA TCT TAT TTA GAA AAA AGG AGA CGT AGA GCC TTG AGT AGG CCT ATT AGA AAA TCC GAA AAC GAC 816

A L V P E E D H D I G S P S Y L E K R R R R A L S R P I R K S E N D 272

GAT CAC TCG GAC TGT TCC TCT GTC TAT TCC CAT CCA TCA ATG CTA GAT TCC GGT GAA ATG TCC TAC CAA CAC GCT GTG CTC GGC GGG CTT AAA GGA TCC TCG 918

D H S D C S S V Y S H P S M L D S G E M S Y Q H A V L G G L K G S S 306

TCG AGC TCA ATG CGA CGA GTC GCC TCG AAT CAG AAG ATC CAA CTT CCC CAG TCA CCA CTA TTC TCG TCA ACT CCA GTC GAC TCG ACG ATC CTC GAT CAA GTA 1020

S S S M R R V A S N Q K I Q L P Q S P L F S S T P V D S T I L D Q V 340

AAG ATC GGA AAG GCG AAT AGT CGA TTG GAT TTC GTG CAA ACT GTG TCG GAT GTG GCT CCG GAT GCT CCG CCA GGC TAT CAG CCA CTA GTT ATA TCC AGA AGG 1122

K I G K A N S R L D F V Q T V S D V A P D A P P G Y Q P L V I S R R 374

ATT CGG CGG CCC TCG AAA CAG AAG TCA AAT TCA AGA AGT TCC AGT GAA TCG GCT TCC AGG GAT AGT CGG CGT GTT ACA GAA GCC ATT TCG ACA CGG GAT TCC 1224

I R R P S K Q K S N S R S S S E S A S R D S R R V T E A I S T R D S 408

AGG GCG AAT AGT GAT TTG CCC TCC ACA ATC TTC GGA ACC GTC CCA AAT GAC GTG GCA TTC TCA CCG GAT GAC GAG CTG CTT CAG CTT CAA TCC AAG CCT ATT 1326

R A N S D L P S T I F G T V P N D V A F S P D D E L L Q L Q S K P I 442

TCC ACG ACA ATT GAC AGT GGA ATT GCT GAA ATG ACG TCA TCA TCG GCT GCC GGA ACC CGT ACA GCT TCA GAA GTG GAG CCA GAA GGA GAG CTC CAA GTT GAG 1428

S T T I D S G I A E M T S S S A A G T R T A S E V E P E G E L Q V E 476

CGT TCC GTC TCG TTT TTG GAA GCT CTT CAC GAG TTG GCT GGA GCT AGA GGA GCT AGA GAC GGC GCG GAG GAA GAA GCC ATT CCA CCA CCC AGA AGC CTA TCG 1530

R S V S F L E A L H E L A G A R G A R D G A E E E A I P P P R S L S 510

CTT ACC GAT GAT TTT TTG GGG AAA AAT GAG GAA AAA TCG GAG CAT GAG GAT GAG GAT CGA GAG ATT TTT GAT GTA TCA ATG GAC CCC GTC GAA GGC ACT TCC 1632

L T D D F L G K N E E K S E H E D E D R E I F D V S M D P V E G T S 544

ATA ACG ATT TTG GCG CCA AAT CCA CCC ACC GGA GAG CTT AAT CGG AGC CAA CCA ATG TCG ATA CCG GGC GGC CGC CGG CAA CTA ATA CAC AGA CCG CAA AAG 1734

I T I L A P N P P T G E L N R S Q P M S I P G G R R Q L I H R P Q K 578

ACC GAC ACA ATT CTC GGC ACC TCA CTC CGC GAT GAG CTC CTC GAG GCA CTC CCA TCA ACG CAC AAT GAC ACA TTT GAG AAT GGC ACC CCG ATC TAC GTA CAA 1836

T D T I L G T S L R D E L L E A L P S T H N D T F E N G T P I Y V Q 612

AGC CCC GGG CTC ATG GGA AAC TCG CTT GCC GCC ATC GGA GCC CGC CAT CAG ATG TGG GAG TCC ACT TCG GAT CGG CTC TCC TCG TCT TCA GAC TCA TGC GGC 1938

S P G L M G N S L A A I G A R H Q M W E S T S D R L S S S S D S C G 646

GGC CCG GTG GTG AGC TTC GGA CAG GCT CTA AGA TCT GCG ATG GAG ACG AGA GAC GAT CCT GAG TCG CTT GGC ACA TCG AGT CAG GAT CTT GCC GAT GTG GAT 2040

G P V V S F G Q A L R S A M E T R D D P E S L G T S S Q D L A D V D 680

GAG CAG GAT GTA GCG GAT TCC TCG CGA AAA TCG TCG GTT TCT GCG GAA ACC GCC GAG AAA TTG TGC ACG ATA CCC AGG GAG AAG GGT CTC GAC GCG CAG GAT 2142

E Q D V A D S S R K S S V S A E T A E K L C T I P R E K G L D A Q D 714

TTT CGG TGT GCC ATG TGT CGG AAA ACG ATT GGT GGA AGC ACG TTT TCC AAG TTT GAA ACC TGC GCC ATC GAC AGC AAA TAC TAC TGC ACA GAA TGT ATG AAG 2244

F R C A M C R K T I G G S T F S K F E T C A I D S K Y Y C T E C M K 748

TCC GGC GGA AAA GTC TCG ATT CCG GCC CGC GTC GTA ATG GAC TGG GAT TGG CGG GAA CGG GCC GTC TCG GAC CGA GGA CGT GCC TGG TAT GAG GCG AAT CAG 2346

S G G K V S I P A R V V M D W D W R E R A V S D R G R A W Y E A N Q 782

GAG AAG GCG CTG ATC AAT ATC AAG ACA ACA AAT AGC CGA CTG TAT GCA CAT GCT CCG GCT CTC GAA GAG ACT CGG AAG CTT CGC GAG AAG CTT CAG CTC GTC 2448

E K A L I N I K T T N S R L Y A H A P A L E E T R K L R E K L Q L V 816

TCA ATG TAC CTA TTC ACG TGC CGG GAA TCT GTG TCA GAG GAC TTT CGG CGT CGA TTG TGG CCT AAA GAG TAT TTG AGA TCG GAA ATT GAC GTG TAT TCC TTT 2550

S M Y L F T C R E S V S E D F R R R L W P K E Y L R S E I D V Y S F 850

T in *cd32*

GCT GAC TTG ATT GAT GTG AAG AGT GGA GCA CTG CAG AGA CGA TTG AAT AGT CTC TTG AAG CAC TCG ATC AAT CAT GTC ATG ACA TGC ACA TTG TGC AAG CAG 2652

A D L I D V K S G A L Q R R L N S L L K H S I N H V M T C T L C K Q 884

* in *cd32*

AAA GGC TTC TGT TGT GAG CTG TGC ACT GTC AAT GAG GTC ATT TAT CCG TTT AAT ACG GAA TCC ACG CAT AGG TGC CTC GTA TGC TTT TCG GCG TTC CAC GTG 2754

K G F C C E L C T V N E V I Y P F N T E S T H R C L V C F S A F H V 918

GAA TGC TGG CGA ACG TCT GGC GAC TGC CCG AAA TGT GTC CGC CGC CAG AAT TTT GAG ACT CGC CGA GCA CAA GTC GAC GAT CCA CAT AAC ACA CTG CTC GTT 2856

E C W R T S G D C P K C V R R Q N F E T R R A Q V D D P H N T L L V 952

CTT CAG CCA TAA 2868

L Q P * 955

**Figure S3** Predicted Open Reading Frame of *cup-14*. The RUN domain is highlighted in pink; the DUF4206 domain is highlighted in red. Changes to the DNA and the protein sequences in *cup-14* alleles are indicated.
