# Supplementary material for: Regulators of Lysosome Function and Dynamics in Caenorhabditis elegans
Source: G3 (Bethesda). 2017 Jan 24;7(3):991–1000. doi: 10.1534/g3.116.037515 (PMC5345728; doi:10.1534/g3.116.037515)
Supplement: Supplementary file 4 [file 991FigureS4.docx]

***F42A8.3* predicted open reading frame ATG-STOP, and 5’ upstream, and first two introns**

gctagaaaatgctttctttttaataaaaattattttgcag ATG GTA AAT TCA CTT TCA AGG ATA CTT TTC TGC AGC TTA CTT ATA TTT TCC GTG ATT TCT CTT CGA GAA GAG 112

M V N S L S R I L F C S L L I F S V I S L R E E 24

Underlined sequence deleted in *cd46*

CGG CTC GAA ACA GCG CCG ATC ACT GAT GAT CCT TGG GAT TTG GAT GGA CCC TGT CAA AAA TAT GTG GAA gtaagattcttcgaagtatttaatgcaactaaacatctttatttcag 228

R L E T A P I T D D P W D L D G P C Q K Y V E 47

AAA CTA GCT GTT GTA CAA TCG GAA ATG GTA GCA TGC GCC ACA AAC TGG TCA ATT CCA CCT GTA GTT TGC ACA AAA TGC TTT CAA AAC TAC ATT AAT TTC AAA CAA 333

K L A V V Q S E M V A C A T N W S I P P V V C T K C F Q N Y I N F K Q 82

TTT GAA TAT GAA ACA AAA AAC CTG gtgagtataatttgaaaacaatattttttaaaaccgtcttcacataaaatatgaattttattacag AAT AAT GTC TAC TCC CTG GAT AAT CGA ACT 453

F E Y E T K N L N N V Y S L D N R T 100

TGC TCT CAA GTT ATT TAT GAC AAT TAT CTG CTC TCG TAC AGT ACG GAT ATA TCA AAA GCT TTA ACT TCA GAA ATT TGG GAA AAA TCC AGA TGT GAC TCC TGC ATA 558

C S Q V I Y D N Y L L S Y S T D I S K A L T S E I W E K S R C D S C I 135

ACA ATT AAA TGG AAT TTT CCG CAA AAC AAA TCG GAA GTG TCA TTC AGT GAG AGG ACA ATG CAA TTT CAG AAC AGA ATG TAT GAA TGG AGA AAT TGT GTT GTT AAT 663

T I K W N F P Q N K S E V S F S E R T M Q F Q N R M Y E W R N C V V N 170

TAC ACA TCT GGA GGT GTA CTG GAT GAT AAT CTT ACC AAT GGA AGT AAA ATC TGT AAT CTC TGC AAG ACC ACC TTT GAC GAA CTC TTT GGA TAT TAC TGG AAA ATC 768

Y T S G G V L D D N L T N G S K I C N L C K T T F D E L F G Y Y W K I 205

A in *cd33*

TAC ACA ACT CCA GAT GTA GAT TTT TGT GTC GAC GTG GAG ACT ACG ATG AAC GAT ACA ATT CAT TTA TGG GAT GAT GTG TGG AAA TGT GCT GAA AAA CAA GAC AGA 873

Y T T P D V D F C V D V E T T M N D T I H L W D D V W K C A E K Q D R 240

* in *cd33*

AAT CGT GAT TTA TTT GGA ATT ATG ATC ACT TTT GGA ACA TTA TTG CTG CTC ACA GCT CTA TTT TAT GCT GCC AGC TAC ATT CAG GGT GGC GGT GAA ACG AGG CGT 978

N R D L F G I M I T F G T L L L L T A L F Y A A S Y I Q G G G E T R R 275

CTT ATT CAA TAT GCT CGT CTA TCA GAT CCA CAT GGC CAA AGA TCA CGT CTT CTT TCA TCG GGA ATG TCG GAT GCA GAT CTT GTT AGT CGT GTT TCT CCA GGA TCT 1083

L I Q Y A R L S D P H G Q R S R L L S S G M S D A D L V S R V S P G S 310

TCT GTG TTA TAC AAT GTA CCA ATC CAT CAG ACA CGA TAA 1122

S V L Y N V P I H Q T R * 322

**Figure S4** Predicted Open Reading Frame of *cup-15*. The transmembrane domain is highlighted in yellow. Changes to the DNA and the protein sequences in *cup-15* alleles are indicated.
