# Supplementary material for: Regulators of Lysosome Function and Dynamics in Caenorhabditis elegans
Source: G3 (Bethesda). 2017 Jan 24;7(3):991–1000. doi: 10.1534/g3.116.037515 (PMC5345728; doi:10.1534/g3.116.037515)
Supplement: Supplementary file 5 [file 991FigureS5.docx]

***C34C6.7* predicted open reading frame ATG-STOP**

ATG ATT TGT TGG CTC AAT TTA TCG ATA TTT TAT TTT GTA TTC TCT CTG TAT CAA TGT CAT CAA GAA GAA TTA TCA CAA AAT ATA GAA GTC ACT GCA TCT GAT 102

M I C W L N L S I F Y F V F S L Y Q C H Q E E L S Q N I E V T A S D 34

TCT GCA GCA GAA CAT GAA GTG TTT GAA GGA ATA TCA TCA AAT ATT GCT GGG AAA GGA GAA AAG TTA GAA GAA GAA ATA GAT AAC ATT GGT ATT GTA ATG CAA 204

S A A E H E V F E G I S S N I A G K G E K L E E E I D N I G I V M Q 68

CCA GAG CCA CGA GTT GTC CAT GAA GCT TCC GAA GTA TCA GAC AAC ATT GAA CTT AAT ATC AAA GAC GAC CTA AAC TTG AAA AGT AGA CTG GAC AAC TTC ACA 306

P E P R V V H E A S E V S D N I E L N I K D D L N L K S R L D N F T 102

AGA GCT AAA TTC AGG CAA TCA ACC ACC GTA ACA CCT AAT ATT GTT GCT GTG GAG CCT TCT ATT GAA GGT GTT GAA GAC GAT TTA GAT CAT GAT GAA CAA GGA 408

R A K F R Q S T T V T P N I V A V E P S I E G V E D D L D H D E Q G 136

GAA CCA GAA GAC AGT GAA ATT CGA AAT CGG AAC GAG CAC CAT TTT GCT GAG TTG GGC GGA AAA ATG AAG GAA CGC AGG GAT CAT GTA GAT GAT CCC GAT ATT 510

E P E D S E I R N R N E H H F A E L G G K M K E R R D H V D D P D I 170

CAA GAA CCA TTG AAT AAG CCA ATA TCG GCT ATT CCA GAA GAG ACT TCA AAT TCT GCA ACT ATT CAA TAT GAA AGA AGT TCG TCT GGA ACA CAA AAC CCG CAA 612

Q E P L N K P I S A I P E E T S N S A T I Q Y E R S S S G T Q N P Q 204

ACT TCT TTA AAT ATT CCG ACT GCT CAG AAT GTA CCA AAA CTA AAA CAA TTA AAA AAT CAA GAG ATT CTA AAG GAT CAT TTT ATT TCG GAA GTA TCA GAT GTT 714

T S L N I P T A Q N V P K L K Q L K N Q E I L K D H F I S E V S D V 238

TCC AAC GTT GCA GAT GTT TCA GCG CCT ATT GTT ATT AGT CAA GTA CCT GAA ATA TTG AAT GGA GAA CCC GCA GGA GTA CCT GCC AAT TTT GAA GAA GAA GAC 816

S N V A D V S A P I V I S Q V P E I L N G E P A G V P A N F E E E D 272

AAA GAA CGT GTA GAA GAA GAG GAA GAT CGC ATC AGT TGG GAT CTT ATT CAT TTT CTA CTT TTG TTA AGT CCA TAT GAA GAA GGT GAA GTT ACT TGG TGG ACA 918

K E R V E E E E D R I S W D L I H F L L L L S P Y E E G E V T W W T 306

T in *cd50*

ATT GTT CTT GAA GCC GTT AAG TGC TCT CTT CGA AGT TGT CAT AAT TCA TCA TCT CAC TGG CAT GAT AGA TCT GTG TAT CTT CCG AGA ATA ACT CGA CAT CGT 1020

I V L E A V K C S L R S C H N S S S H W H D R S V Y L P R I T R H R 340

L in *cd50*

CGT CAA GAT TCA GAC TAT TTA CCT GGA GCA CTT TCT CCA ACA ACA CCA AAA CCC TGT TTG TGC AAC AAT TTA GAG ACT TAT TTC GAA AAA CTG TAT GAA TCA 1122

R Q D S D Y L P G A L S P T T P K P C L C N N L E T Y F E K L Y E S 374

GTT GGG AAA AAA AAG AAG GAA AAG AAA ATA TCA AAT TCA ACA ACG AAA TTT GTA TAA 1179

V G K K K K E K K I S N S T T K F V * 392

**Figure S5** Predicted Open Reading Frame of *cup-16*. Changes to the DNA and the protein sequences in the *cup-16(cd50)* allele are indicated.
