# Supplementary material for: Regulators of Lysosome Function and Dynamics in Caenorhabditis elegans
Source: G3 (Bethesda). 2017 Jan 24;7(3):991–1000. doi: 10.1534/g3.116.037515 (PMC5345728; doi:10.1534/g3.116.037515)
Supplement: Supplementary file 6 [file 991FigureS6.docx]

***ctns-1a* predicted open reading frame ATG-STOP**

ATG AGT TTC CCG GTG GCA TTT TTG CTC GTT CTC TTC TTG GTT CCA TTC ACA TTT GCG ACA AAT AAT TTA GTT GTT AGA CAG AAA GAA TTG GAA ATT GTA ATT 102

M S F P V A F L L V L F L V P F T F A T N N L V V R Q K E L E I V I 34

GGA GGA GAA GTC TCT GTT AAT TTC CAG ATC AAA AAC CAC ACA TCA CAA TCA TTG AAC GCA ACA CGT ATC TCC CTC TCG CAG TCA CCA TAC ATC TCC CAT CCT 204

G G E V S V N F Q I K N H T S Q S L N A T R I S L S Q S P Y I S H P 68

GAT GCT ATT CTC GTT GAC AAT TGG AAT GCT AAT GTG ACA GTT TTA GGA AGC CAA TTG GTG TCC GGA GCT ATT TTA GAA GCT CTC AAT TGT ACT ACA GAT GGG 306

D A I L V D N W N A N V T V L G S Q L V S G A I L E A L N C T T D G 102

TCA ATT ACT TGT CCT CTG GAT CTT GAA GAT GCG TTT GCT AGA ATC ACA GTT ATT CGC TCT CAC TTT CTT GCC ATT TTG ATT CAA ATT GTT GGA TGG ACA TAC 408

S I T C P L D L E D A F A R I T V I R S H F L A I L I Q I V G W T Y 136

TTC TTC GCA TGG AGT ATT TCG TTT TAT CCC CAA ATG TAT CTG AAT TTC AAA AGA AAA AGC GTC GTC GGG CTA AAC TTC GAT TTC TTA TCT CTG AAC CTT GTC 510

F F A W S I S F Y P Q M Y L N F K R K S V V G L N F D F L S L N L V 170

GGA TTT TGT GCA TAT GCT ATT TTC AAT CTG TTA ATG TAT TAT AAT AGT CAT GTA AAG AAT GAA TAC AAT ATT GTG AAT CCT CGG TCA CCG CCA CCA GTA CTT 612

G F C A Y A I F N L L M Y Y N S H V K N E Y N I V N P R S P P P V L 204

TTG AAC GAT GTT GTG TTC GCT GTT CAC GCA TTT CTT GCC TGC TTT ATC ACA ATT CTT CAA TGT TTA TTC TAT GAA CGT GAT AAT CAG AGT GTT TCT TCA AAA 714

L N D V V F A V H A F L A C F I T I L Q C L F Y E R D N Q S V S S K 238

TGT ATC GCC CTT ATG ATT GTG CTG ATC TCT TTT GGA TTC TGC TCC GCT GCC GCC ACG GTT CTC AGG AAA ATT CAA TTG CTC TCG TTT GTG ACC AGT TTG TCG 816

C I A L M I V L I S F G F C S A A A T V L R K I Q L L S F V T S L S 272

T in *cd49*

TAC ATA AAA ATG GCT GTA ACC TGC TGT AAA TAT TTC CCA CAG GCT TAC TTC AAC TAT ACC CGA AAA AGT ACA GTT GGA TGG TCA ATC GGA AAC ATC ATG CTA 918

Y I K M A V T C C K Y F P Q A Y F N Y T R K S T V G W S I G N I M L 306

* in *cd49*

GAT TTC ACA GGA GGA ACT CTC GAT ATT CTT CAA ATG ATT CTT CAA GCA GTT AAT GTG AAC GAT TGG TCT GCA TTT TAT GCG AAT CCC GTC AAA TTC GGA CTG 1020

D F T G G T L D I L Q M I L Q A V N V N D W S A F Y A N P V K F G L 340

GGA TTT GTC TCA ATA TTC TTT GAC ATC ATT TTC ATG GTT CAA CAT TAT GTA TTG TAT CCA AAT GCA GAA GAA CCA AAG AAA AAT CAG GAA ACG AGT CGT TTT 1122

G F V S I F F D I I F M V Q H Y V L Y P N A E E P K K N Q E T S R F 374

TAA 1125

*

**Figure S6** Predicted Open Reading Frame of *cup-17/ctns-1*. The transmembrane domains are highlighted in yellow; the CTNS domains are highlighted in blue. Changes to the DNA and the protein sequences in the *cup-17(cd49)* allele are indicated.
