# Supplementary material for: Regulators of Lysosome Function and Dynamics in Caenorhabditis elegans
Source: G3 (Bethesda). 2017 Jan 24;7(3):991–1000. doi: 10.1534/g3.116.037515 (PMC5345728; doi:10.1534/g3.116.037515)
Supplement: Supplementary file 7 [file 991FigureS7.pdf]

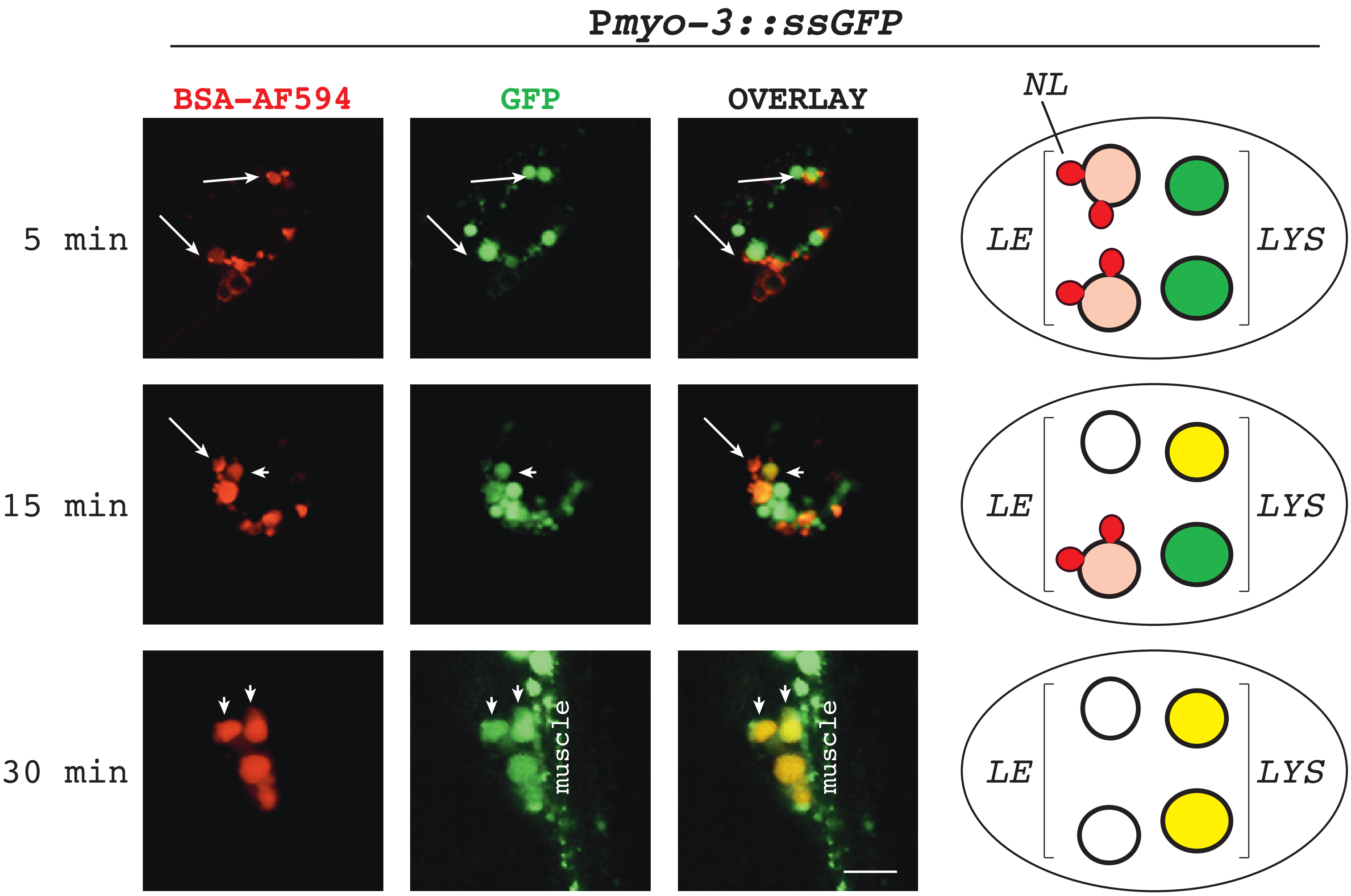

Figure S7 Pulse-Chase Analysis of Endocytosed BSA-Alexa Fluor 594. Left panels are confocal single optical sections of *arIs37[Pmyo-3::ssGFP; dpy-20]* adult hermaphrodites at the indicated times after the microinjection of BSA-Alexa Fluor 594 into their body cavities. Arrows indicate nascent lysosomes containing concentrated BSA-Alexa Fluor 594; arrowheads indicate lysosomes that contain both GFP and BSA-Alexa Fluor 594. Bar is 5 μm. Right panels are a schematic of the BSA-Alexa Fluor 594 pulse-chase analysis. Co-localization of BSA-Alexa Fluor 594 (red) and GFP (green) is shown in yellow. LE =Late Endosome; NL = Nascent Lysosome; LYS = Lysosome.
