# Supplementary material for: Regulators of Lysosome Function and Dynamics in Caenorhabditis elegans
Source: G3 (Bethesda). 2017 Jan 24;7(3):991–1000. doi: 10.1534/g3.116.037515 (PMC5345728; doi:10.1534/g3.116.037515)
Supplement: Supplementary file 8 [file 991FigureS8.pdf]

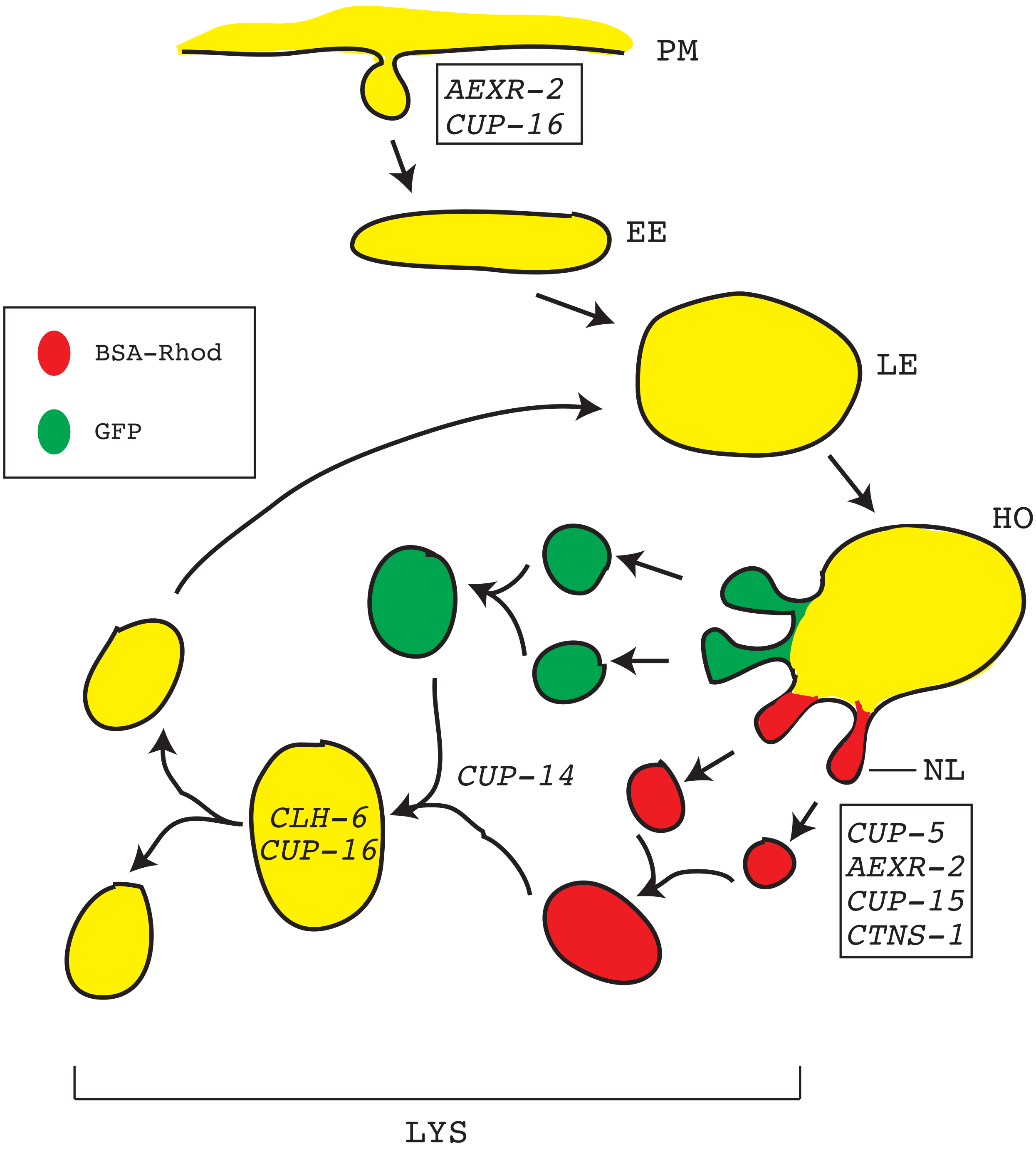

Figure S8 Model of novel CUP functions at the plasma membrane and endosomes. Shown is a simplified schematic of an endocytic pathway with presumed functions for the newly identified CUP proteins. EE = Early Endosome; HO = Hybrid Organelle; LE = Late Endosome; LYS = Lysosome; NL = Nascent Lysosome; PM = Plasma Membrane.
