# Supplementary material for: Regulators of Lysosome Function and Dynamics in Caenorhabditis elegans
Source: G3 (Bethesda). 2017 Jan 24;7(3):991–1000. doi: 10.1534/g3.116.037515 (PMC5345728; doi:10.1534/g3.116.037515)
Supplement: Supplementary file 9 [file 991TableS1.docx]

| **Table S1. *C. elegans* strains used in this study** | |
| --- | --- |
| **STRAIN NAME** | **STRAIN GENOTYPE** |
| GS1912 | *dpy-20(e1282); arIs37[*P*myo-3::ssGFP; dpy-20]* |
| VC20266 | *Y108G3AL.2(gk223891)* |
| VC40681 | *Y08G3AL.3(gk760183)* |
| VC40245 | *aexr-2(gk533021)* |
| VC30127 | *col-168(gk963311)* |
| VC40675 | *F14B8.5(gk758293)* |
| VC40416 | *F14B8.5(gk626793)* |
| VC20585 | *gcy-14(gk254630)* |
| VC20436 | *clh-6(gk248753)* |
| VC40363 | *Y51H1A.2(gk595958)* |
| VC876 | *cpna-2(gk248)* |
| VC20190 | *cpna-2(gk147501)* |
| VC40980 | *srh-60(gk910522)* |
| VC40021 | *srh-60(gk138763)* |
| VC20316 | *W10G11.17(gk138717)* |
| VC20583 | *W10G11.17(gk348016)* |
| VC20488 | *F19B10.1(gk138993)* |
| VC703 | *ani-2(ok1147)/mIn1* |
| VC40053 | *F18A1.1(gk147411)* |
| VC20620 | *C34C6.7(gk358192)* |
| VC2775 | *cct-2(ok3438)/mIn1* |
| VC20554 | *C18E9.9(gk149970)* |
| VC20339 | *C18E9.9(gk149971)* |
| VC20339 | *C18E9.9(gk149971)* |
| VC40082 | *T15H9.6(gk151217)* |
| PS5131 | *let-23(sy12)/mIn1* |
| VC30246 | *T01H3.2(gk446662)* |
| VC20421 | *F42A8.3(gk150714)* |
| RB934 | *ctns-1(ok813)* |
| GS1912 | *dpy-20(e1282); arIs37[*P*myo-3::ssGFP; dpy-20]* |
| GS2477 | *cup-5(ar465); dpy-20(e1282); arIs37[Pmyo-3::ssGFP; dpy-20]* |
| NP1500 | *cup-5(cd9); dpy-20(e1282); arIs37[Pmyo-3::ssGFP; dpy-20]* |
| NP1488 | *cup-5(cd10); dpy-20(e1282); arIs37[Pmyo-3::ssGFP; dpy-20]* |
| NP1397 | *cup-5(cd12); arIs37[Pmyo-3::ssGFP; dpy-20]* |
| NP1324 | *cup-5(cd18); dpy-20(e1282); arIs37[Pmyo-3::ssGFP; dpy-20]* |
| NP1487 | *cup-5(cd19); dpy-20(e1282); arIs37[Pmyo-3::ssGFP; dpy-20]* |
| NP1483 | *cup-5(cd20); dpy-20(e1282); arIs37[Pmyo-3::ssGFP; dpy-20]* |
| NP1484 | *cup-5(cd21); dpy-20(e1282); arIs37[Pmyo-3::ssGFP; dpy-20]* |
| GS2484 | *cup-11(ar472); dpy-20(e1282); arIs37[Pmyo-3::ssGFP; dpy-20]* |
| GS2496 | *cup-11(ar480); dpy-20(e1282); arIs37[Pmyo-3::ssGFP; dpy-20]* |
| GS2498 | *cup-11(ar482); dpy-20(e1282); arIs37[Pmyo-3::ssGFP; dpy-20]* |
| GS2513 | *cup-11(ar487); dpy-20(e1282); arIs37[Pmyo-3::ssGFP; dpy-20]* |
| GS2524 | *cup-11(ar491); dpy-20(e1282); arIs37[Pmyo-3::ssGFP; dpy-20]* |
| GS2541 | *cup-11(ar501); dpy-20(e1282); arIs37[Pmyo-3::ssGFP; dpy-20]* |
| GS2551 | *cup-11(ar502); dpy-20(e1282); arIs37[Pmyo-3::ssGFP; dpy-20]* |
| GS2552 | *cup-11(ar503); dpy-20(e1282); arIs37[Pmyo-3::ssGFP; dpy-20]* |
| GS2559 | *cup-11(ar510); dpy-20(e1282); arIs37[Pmyo-3::ssGFP; dpy-20]* |
| NP1345 | *cup-12(cd7); dpy-20(e1282); arIs37[Pmyo-3::ssGFP; dpy-20]* |
| NP1314 | *cup-12(cd14); dpy-20(e1282); arIs37[Pmyo-3::ssGFP; dpy-20]* |
| NP1315 | *cup-12(cd15); dpy-20(e1282); arIs37[Pmyo-3::ssGFP; dpy-20]* |
| NP1363 | *cup-12(cd34); dpy-20(e1282); arIs37[Pmyo-3::ssGFP; dpy-20]* |
| NP1374 | *cup-12(cd39); arIs37[Pmyo-3::ssGFP; dpy-20]* |
| NP1346 | *cup-13(cd17); arIs37[Pmyo-3::ssGFP; dpy-20]* |
| NP1848 | *cup-13(cd16); arIs37[Pmyo-3::ssGFP; dpy-20]* |
| NP1360 | *cup-14(cd31); arIs37[Pmyo-3::ssGFP; dpy-20]* |
| NP1378 | *cup-14(cd32); arIs37[Pmyo-3::ssGFP; dpy-20]* |
| NP1379 | *cup-15(cd33); arIs37[Pmyo-3::ssGFP; dpy-20]* |
| NP1388 | *cup-15(cd46); arIs37[Pmyo-3::ssGFP; dpy-20]* |
| NP1639 | *cup-16(cd50); arIs37[Pmyo-3::ssGFP; dpy-20* |
| NP1531 | *cup-17(cd49); dpy-20(e1282); arIs37[Pmyo-3::ssGFP; dpy-20* |
| NP1129 | *unc-119(ed3); cdIs131[*P*cc1::GFP::rab-5; unc-119-myo-2::GFP]* |
| NP1869 | *cup-5(ar465); cdIs131[*P*cc1::GFP::rab-5; unc-119-myo-2::GFP]* |
| NP1750 | *cup-12(cd7); cdIs131[*P*cc1::GFP::rab-5; unc-119-myo-2::GFP]* |
| NP1748 | *cup-13(cd17); cdIs131[*P*cc1::GFP::rab-5; unc-119-myo-2::GFP]* |
| NP1816 | *cup-14(cd32); cdIs131[*P*cc1::GFP::rab-5; unc-119-myo-2::GFP]* |
| NP1799 | *cup-15(cd33); cdIs131[*P*cc1::GFP::rab-5; unc-119-myo-2::GFP]* |
| NP1800 | *cup-16(cd50); cdIs131[*P*cc1::GFP::rab-5; unc-119-myo-2::GFP]* |
| NP1711 | *cup-17(cd49); cdIs131[*P*cc1::GFP::rab-5; unc-119-myo-2::GFP]* |
| NP1200 | *unc-119(ed3); cdIs153[rme-8::GFP; unc-119-myo-2::GFP]* |
| NP1870 | *cup-5(ar465); cdIs153[rme-8::GFP; unc-119-myo-2::GFP]* |
| NP1757 | *cup-12(cd7); cdIs153[rme-8::GFP; unc-119-myo-2::GFP]* |
| NP1749 | *cup-13(cd17); cdIs153[rme-8::GFP; unc-119-myo-2::GFP]* |
| NP1796 | *cup-14(cd32); cdIs153[rme-8::GFP; unc-119-myo-2::GFP]* |
| NP1758 | *cup-15(cd33); cdIs153[rme-8::GFP; unc-119-myo-2::GFP]* |
| NP1802 | *cup-16(cd50); cdIs153[rme-8::GFP; unc-119-myo-2::GFP]* |
| NP1724 | *cup-17(cd49); cdIs153[rme-8::GFP; unc-119-myo-2::GFP]* |
| NP745 | *unc-119(ed3); cdIs40[*P*cc1::GFP::cup-5; unc-119-myo-2::GFP]* |
| NP1754 | *cup-12(cd7); cdIs40[*P*cc1::GFP::cup-5; unc-119-myo-2::GFP]* |
| NP1755 | *cup-13(cd17); cdIs40[*P*cc1::GFP::cup-5; unc-119-myo-2::GFP]* |
| NP1798 | *cup-14(cd32); cdIs40[*P*cc1::GFP::cup-5; unc-119-myo-2::GFP]* |
| NP1752 | *cup-15(cd33); cdIs40[*P*cc1::GFP::cup-5; unc-119-myo-2::GFP]* |
| NP1801 | *cup-16(cd50); cdIs40[*P*cc1::GFP::cup-5; unc-119-myo-2::GFP]* |
| NP1699 | *cup-17(cd49); cdIs40[*P*cc1::GFP::cup-5; unc-119-myo-2::GFP]* |
| NP1580 | *cup-5(zu223) unc-36(e251)/qC1; pwIs50[lmp-1::GFP; unc-119]; kxEx148(F11E6.1a::mCherry; rol-6(su1006)]* |
